# Supplementary material for: Cooperative adsorption of carbon disulfide in diamine-appended metal–organic frameworks
Source: Nat Commun. 2018 Dec 3;9:5133. doi: 10.1038/s41467-018-07458-6 (PMC6277438; doi:10.1038/s41467-018-07458-6)
Supplement: Supplementary file 1 — Supplementary Information [file 41467_2018_7458_MOESM1_ESM.pdf]

Supporting Information

Cooperative Adsorption of Carbon Disulfide in Diamine-Appended Metal–Organic Frameworks

McGuirk, et al.

Supplementary Methods

Ex Situ FT–IR Measurements

Infrared spectra were collected using a PerkinElmer Avatar Spectrum 400 FT–IR spectrophotometer in transmittance mode using KBr windows. For each measurement, ~10 mg of sample was ground with an agate mortar and pestle with 4–5 drops of Nujol light mineral oil from Sigma-Aldrich, and subsequently transferred to the KBr windows with a metal spatula. Samples were pre-activated at 80 °C under flowing N_2 for 2 h. Spectra of CS_2-dosed samples were prepared using material that had been used for 25 °C CS_2 isotherms, which included desorption down to 1 mbar (as shown in Supplementary Figure 24), and subsequently exposed to dynamic vacuum (~0.13 mbar) at 25 °C for 16 h. Reactivated spectra were of CS_2-dosed samples that were subsequently exposed to dynamic vacuum (~0.13 mbar) at elevated temperatures, as specified in respective figure captions.

Thermogravimetric Analysis of CS_2-Dosed mm-2–M_2(dobpdc) (M = Mg, Mn)

Thermogravimetric analyses (TGA) were conducted using a TA Instruments TGA Q5000 with a flow rate of 25 mL min^{-1} for all gases. Masses were uncorrected for buoyancy effects. For control isobars under 100% CO_2 at 1 bar, samples were first activated under flowing N_2 at 120 °C for 30 min. The gas was then switched to 100% CO_2 and the mass normalized to 0. Cooling rates of 2 °C min^{-1} were used for isobars. CS_2-dosed samples were prepared using material that had been used for 25 °C CS_2 isotherms, which included desorption down to 1 mbar (as shown in Supplementary Figure 29). For CO_2 isobars of CS_2-dosed samples, samples were re-activated under flowing N_2 at the temperature and for the time duration noted in respective figure caption. The gas was then switched to 100% CO_2 and the mass normalized to 0. Cooling rates of 2 °C min^{-1} were used for all isobars unless otherwise specified. Of note, mm-2–Zn_2(dobpdc) does not show CO_2 adsorption under 1 bar of CO_2 above 30 °C, and therefore isobaric measurements on our instrument, which does not have the ability to cool, were not informative in probing the cooperative adsorption behavior of this material.

CS_2 Adsorption–Desorption Cycling

The adsorption–desorption cycling experiment was prepared similarly to as described above for CS_2 isothermal adsorption measurements. Temperature was toggled using a silicone oil bath on a hot plate possessing a thermocouple probe. The temperature of the bath was cross-referenced with a secondary external digital temperature probe. Adsorption equilibrium was assumed when the variation of pressure was 0.01% or lower over 240 seconds. The bath was cycled between 35 °C and 100 °C. Upon changing the temperature of the oil bath, the bath and sample sat at the given temperature for 30 minutes to allow thermal equilibrium to occur. Adsorption capacity at 75 mbar of CS_2 was measured for each successive data point.

CS_2 dosing of mm-2–Zn_2(dobpdc) single crystals and analysis by X-ray diffraction. CCDC #: 1834399

Single crystals of mm-2–Zn_2(dobpdc) were synthesized and washed as reported previously.[2] Separately, a CS_2 vapor-dosing chamber was prepared by adding approximately 2 mL of CS_2 to a 20 mL scintillation vial. The vial was sealed and allowed to stand at room temperature for ~1 h to saturate the vial atmosphere with CS_2 vapor. Several toluene-washed single-crystals of mm-2–Zn_2(dobpdc) were then transferred by pipette to a 4 mL glass vial in air. Excess toluene was

removed by pipette, and the 4 mL vial was then placed inside the 20 mL CS_2 vapor chamber. The vapor chamber was sealed and kept at room temperature. After 10 min, the inner 4 mL vial was removed, and the crystals were covered with paratone oil. The vial was then sealed in air, and the CS_2-dosed crystals were analyzed within 24 h of preparation.

Single-crystal X-ray diffraction data were collected at Beamline 11.3.1 at the Advanced Light Source, Lawrence Berkeley National Laboratory using synchrotron radiation (λ = 0.7749 Å) and a Bruker AXS D8 diffractometer equipped with a Bruker PHOTON 100 CMOS detector. Structures were collected at 100 K using an Oxford Cryosystems Cryostream 700 Plus. The structure of CS_2-inserted mm-2–Zn_2(dobpdc) was refined as an inversion twin in space group $P3_121$ based on a Flack parameter value near 0.5. Raw data were corrected for Lorentz and polarization effects using Bruker AXS SAINT software[3] and were corrected for absorption using SADABS.[4] The structures were solved using SHELXT and refined using SHELXL[5] operated in the OLEX2[6] interface. Thermal parameters were refined anisotropically for all non-hydrogen atoms. All hydrogen atoms were placed geometrically and refined using a riding model.

The structure shows CS_2 inserted into the Zn–N bonds of mm-2–Zn_2(dobpdc) to form chains of ammonium dithiocarbamate. The ammonium dithiocarbamate chains were found to be disordered over two positions from the sulfur bound to Zn (S1), requiring distance (SADI for C–N bonds) and displacement parameter (RIGU) restraints. Refinement of each of the disordered residues resulted in chemical occupancies of 47.5(7)% (part -1, atoms with suffix A in the cif) and 39.9(7)% (part -2, atoms with suffix B in the cif), which were fixed in the final refinement. The overall ammonium dithiocarbamate occupancy is 87.4%; however, the sulfur atom bound to Zn (S1) was kept at fully occupancy to account for solvent, water, CS_2, or unreacted diamine that is bound on sites where the chains are absent. The reported formula, however, reflects the sulfur content for the ammonium dithiocarbamate chains alone.

Powder X-ray Diffraction Analysis of CS_2-Dosed mm-2–Mn_2(dobpdc) and mm-2–Mg_2(dobpdc)

Our attempts to grow single crystals of mm-2–Mn_2(dobpdc) and mm-2–Mg_2(dobpdc) resulted in samples that were not amenable to single-crystal X-ray diffraction analyses. Both samples, however, exhibited relatively high crystallinity, allowing them to be studied by powder X-ray diffraction (PXRD). Powder samples were packed into borosilicate glass capillaries of 1 mm diameter and dosed with 50 mbar of CS_2 gas pressure at 25 °C using a Micromeritics 3Flex surface characterization instrument and allowed to equilibrate for one hour. The capillaries were subsequently evacuated under reduced pressure at 25 °C and flame sealed. High-resolution PXRD patterns of the CS_2-inserted samples were collected with synchrotron X-ray radiation at Beamline 17-BM-B, at the Advanced Photon Source of Argonne National Laboratory, at an average wavelength of 0.45241 Å. Scattered intensity was recorded by a PerkinElmer a-Si flat panel detector, while the samples were kept at 100 K using an Oxford Cryosystems Cryostream 800.

The analysis of the diffraction data was performed with the TOPAS 4.1 program.[7] Initially, the powder patterns were analyzed by a structureless Pawley fitting,[8] confirming that all diffraction peaks can be explained using the trigonal space group $P3_121$, with similar unit cell dimensions as those observed for the CS_2-inserted mm-2–Zn_2(dobpdc) crystal structure, solved by single-crystal X-ray diffraction. Rietveld refinements[9] of the CS_2-inserted mm-2–Mn_2(dobpdc) and CS_2-inserted

mm-2–Mg_2(dobpdc) crystal structures were performed using the single-crystal structure of CS_2-inserted mm-2–Zn_2(dobpdc) as a starting model, after replacing the Zn^{2+} with Mn^{2+} and Mg^{2+}, respectively. The starting values of the lattice parameters were extracted from the corresponding Pawley fittings. The lattice parameters, profile parameters (strain, stress, instrumental parameters, simple axial model parameter and zero-error shift), background parameters (Chebyshev polynomial of 10th order) and site occupancies of the dithiocarbamate moieties were freely refined. Preferred orientation of the powder particles was detected in both patterns along the [**001**] direction, and successfully treated with a single March-Dollase parameter (which resulted in a decrease of the R_{wp} figure-of-merit of less than 2%). The refinements converged quickly with satisfactory figures-of-merit.

The crystal structure of CS_2-inserted mm-2–Mn_2(dobpdc) refined with the following unit cell parameters: $a = b = 21.787(3)$ Å, $c = 7.05(2)$ and $V = 2898.9(5)$ Å^3, and figures-of-merit: $R_{exp} = 1.16\%$, $R_{wp} = 6.6\%$, $R_{Bragg} = 1.2\%$. Both conformations were detected with 40% and 37%, similar to the distribution in the CS_2-inserted mm-2–Mn_2(dobpdc) crystal structure. The final Rietveld plot is given in Supplementary Figure 50.

The crystal structure of CS_2-inserted mm-2–Mg_2(dobpdc) refined with the following unit cell parameters: $a = b = 21.548(4)$ Å, $c = 6.89(9)$ and $V = 2773.4(6)$ Å^3, and figures-of-merit: $R_{exp} = 1.28\%$, $R_{wp} = 7.2\%$, $R_{Bragg} = 1.7\%$. Only the less sterically encumbered mode was detected with 30%, while the occupancy of the other mode refined closed to zero. It should be noted, however, that other modes might still be present in the crystal structure, but they were not detected in our analyses due to the pronounced dynamic and conformational disorder of the molecules and/or their low fractional occupancies. Moreover, the Mg sample exhibits low crystallinity (which is visually apparent in comparison with the Mn sample) that further hinders detailed analyses. The final Rietveld plot is given in Supplementary Figure 51.

DRIFTS Studies

Infrared spectra were collected using a Bruker Vertex 70 spectrometer equipped with a glowbar source, KBr beamsplitter, and a liquid N_2 cooled mercury-cadmium-telluride detector. A custom-built diffuse reflectance system equipped with a sample cell which allows control of the atmosphere in the sample chamber through an attached vacuum manifold was used for all experiments. A stainless-steel bomb containing approximately 10 mL of dry CS_2, which had been subjected to three freeze–pump–thaw cycles, was attached to the manifold and isolated. In a typical experiment, pre-activated (16 hours, 80 °C, dynamic vacuum) powder samples of the frameworks (~40 mg) were transferred into the chamber, and the cell was held under vacuum at 25 °C for 16 hours. Dosing was performed by allowing CS_2 vapor from the steel bomb to expand into the evacuated manifold and sample cell. Spectra were collected continually in 60 second intervals at a resolution of 4 cm^{-1} until changes ceased.[10,11]

DFT Vibrational Calculations

DFT geometry and vibrational calculations were performed on the Tiger cluster at the Molecular Graphics and Computation Facility at the University of California, Berkeley using Gaussian 09 paired with Gaussview 5. Truncated single metal site analogs of CS_2-dosed mm-2–Mg_2(dobpdc), mm-2–Mn_2(dobpdc) and mm-2–Zn_2(dobpdc) were built (Supplementary Figures 56–57). The structures were geometrically optimized in the gas phase using M11–L functional[12] along with the Stuttgart/Dresden ECPs (SDD) as implemented in Gaussian[13]. From the

geometrically optimized structures, vibrational frequencies calculations were similarly performed using M11–L/SDD in the gas phase at STP default settings (1 atm, 298.150 K).

NEXAFS Studies

Experimental Details

Nitrogen K-edge NEXAFS measurements were performed at beamline 6.3.2 at the Advanced Light Source using a custom-built gas cell and similar apparatus as previously described[14-16]. Samples were prepared by suspending and sonicating about 10 mg of mm-2–Mg_2(dobpdc) or mm-2–Zn_2(dobpdc) in hexanes and drop-casting onto 300 nm thick silicon carbide membranes (NTT Advanced Technology Corp.). Drop-cast samples were then activated under dynamic vacuum (~0.13 mbar) in a glass chamber with heating at 100 °C for 16 h. Activated samples were kept under nitrogen and loaded into the gas cell in a nitrogen glove box where it was sealed before transporting to the beamline. Activated spectra were collected in vacuum before any exposure to CS_2. CS_2 vapor was subsequently introduced into the gas cell for 1 hour, allowing full adsorption equilibration to occur. Excess CS_2 was then pumped out of the gas cell for 30 minutes before spectra of CS_2-dosed sample was collected. A blank silicon carbide window was used for background correction. For normalization, a line was regressed to the pre-edge region and a polynomial regressed to the post-edge region using the Athena software package[17]. The sample position was not moved during measurement to minimize effects due to spatial inhomogeneity of the sample.

Sulfur K-edge NEXAFS measurements were carried out at the bending magnet microprobe beamline 10.3.2 of the Advanced Light Source, Lawrence Berkeley National Laboratory[18]. Only CS_2-inserted mm-2–Zn_2(dobpdc) and mm-2–Mg_2(dobpdc) were measured as activated materials contain no sulfur. Samples were prepared by suspending and sonicating about 10 mg of mm-2–Mg_2(dobpdc) or mm-2–Zn_2(dobpdc) in hexanes and drop-casting onto 150 nm thick silicon nitride membranes (Silson Ltd). Drop-cast samples were then activated under dynamic vacuum (~0.13 mbar) in a glass chamber with heating at 100 °C for 16 h. The activated samples were then pre-dosed with CS_2 vapor under N_2. A second silicon nitride membrane was then placed on top and sealed with Torr Seal® epoxy in a glovebox to prevent exposure of the MOFs to air. Micro X-ray fluorescence (μXRF) S distribution maps were collected on the samples, then sulfur K-edge NEXAFS spectra were recorded at various sample locations in fluorescence mode using an Amptek silicon drift fluorescence detector 1-element (XR-100SDD). Spectra were collected at ambient temperature, in an energy range of 2430–2560 eV, in QXAS mode where the Si (111) monochromator is scanned continuously during data collection. Each sample spectrum shown is an average of the spectra collected at 14 different locations on the sample. Spectra were calibrated with the sulfur K-edge main peak of gypsum taken at 2482.75 eV. Data reduction and normalization was done using the custom LabVIEW software developed at beamline 10.3.2 and the Athena software package[17].

Structural optimizations

The structures of mm-2–Mg_2(dobpdc) and mm-2–Zn_2(dobpdc) are fully optimized using the Vienna Ab Initio Simulation Package (VASP)[19,20]. The Perdew-Burke-Ernzerhof (PBE) type generalized gradient approximation is used to approximate the exchange-correlation energy in DFT[21,22] and the projector-augmented wave potentials are used to replace the all-electron atomic potentials.[23,24] The plane-wave basis set is truncated at 400 eV and a k-point grid of 1×1×3 is used to sample the first Brillouin zone of each unit cell. It is known that standard local or semi-local exchange-correlation functionals, such as LDA or GGA, may fail to capture weak intermolecular

interactions within the MOF structures due to the absence of the description of long-range dispersion forces. In Supplementary Table 2, we compare the structural parameters obtained for mm-2–Mg_2(dobpdc) and mm-2–Zn_2(dobpdc) using exchange-correlation functionals that are semi-local (PBE), non-local (vdW-DF2[25]), and empirically corrected (the D2 method of Grimme[26]). For the CS_2-dosed mm-2–Zn_2(dobpdc) structure, PBE, vdW-DF2 and D2 methods all provide good agreement with the experiment. For the activated mm-2–Zn_2(dobpdc) structure, both vdW-DF2 and D2 methods fail to reproduce the Zn–O coordination environment and as a result the Zn–Zn distances are much more expanded as shown in Supplementary Table 2. For the mm-2–Mg_2(dobpdc) structure, we do not have the experimental single crystal structure to compare with; however, all PBE, vdW-DF2 and D2 methods produce similar results. For the remainder of this work, we used the standard PBE functional to calculate the structures of mm-2–Mg_2(dobpdc) and mm-2–Zn_2(dobpdc).

Molecular dynamics

To sample the molecular motions at finite temperatures, we carried out *ab initio* molecular dynamics simulations[27] for both mm-2–Mg_2(dobpdc) and mm-2–Zn_2(dobpdc) in which the simulation cells are doubled along in the *c*-axis. The system was equilibrated at 300 K using a Nosé thermostat[28] employing a time step of 0.25 fs and a production run of 3 ps, from which five representative structures were picked with a time interval of 0.5 ps to compute the nitrogen K-edge X-ray absorption spectrum.

NEXAFS simulations

The X-ray absorption spectra based on the structures obtained above are calculated using the PWSCF code within the Quantum-ESPRESSO package[27], where the standard PBE-GGA functional is used with ultrasoft pseudopotentials[28]. A kinetic energy cutoff of 25 Rydberg is used to truncate the plane-wave representation of the electronic wave functions and a reduced k-point mesh of 1×1×2 is used in all supercell self-consistent-field calculations. Transition amplitudes are calculated according to Fermi's golden rule for X-ray absorption cross-sections.[29] Initial states are the 1s orbitals of the N or S atoms and final states are the accessible, or unoccupied, Kohn-Sham eigenstates determined from a self-consistent field calculated within the eXcited electron and Core Hole (XCH) approach.[30] Each computed transition was convoluted with a 0.2 eV Gaussian line shape to produce a continuous spectrum. Nitrogen K-edge simulations were performed on all five representative structures from molecular dynamics, and averaged. Sulfur K-edge calculations used one molecular dynamics structure as the metal–S interactions are fairly rigid and adding more dynamics does not qualitatively change the spectrum. The PBE–GGA functional is known to underestimate band gaps and/or band width,[31-34] leading to spectra that may be compressed along the energy axis. A dilation factor of 1.25 was applied to all nitrogen K-edge calculations and a dilation factor of 1.3 to sulfur K-edge calculations. Three-dimensional isosurfaces of electronic final states are based on core-excited electronic orbitals arising from one core hole and an excited electron in the self-consistent field. These isosurfaces allow for correlating specific transitions to unique chemical bonding. A discrepancy is apparent between the energies of the pre-edge features in the simulated and measured nitrogen K-edge spectra. This may be due to these PBE calculations not accurately describing the highly localized π^* states in the system. However, both mm-2–Mg_2(dobpdc) and mm-2–Zn_2(dobpdc) show nearly the same changes in the experimental NEXAFS spectra upon CS_2 adsorption, and independently the computed spectra of the insertion structure for both MOFs are quite similar to one another. Furthermore, the DFT-computed spectra of previously studied CO_2 adsorption in 2°/2° *N,N′*-dimethylethylenediamine–Mg_2(dobpdc) reveals a pre-edge π^* peak from the carbamate that is about 1 eV higher than the predicted pre-edge π^* peak for the

dithiocarbamate structures investigated here. This is expected due to the stronger π-bonding in carbamate compared to dithiocarbamate.

Water Saturation *Ex Situ* FT–IR Measurements

Infrared spectra were collected using a PerkinElmer Avatar Spectrum 400 FT–IR spectrophotometer in transmittance mode using KBr windows. For each measurement, ~10 mg of sample was ground with an agate mortar and pestle with 4–5 drops of Nujol light mineral oil from Sigma-Aldrich, and subsequently transferred to the KBr windows with a metal spatula.

Compiled spectra for mm-2–Mg_2(dobpdc) are found in Supplementary Figure 82. mm-2–Mg_2(dobpdc) was pre-activated at 100 °C under flowing N_2 for 4 h. A control spectrum of activated mm-2–Mg_2(dobpdc) was collected (black trace). Then, activated mm-2–Mg_2(dobpdc) was dosed with CS_2 through vapor diffusion in a sealed vial for *10 minutes* (red trace). Next, activated mm-2–Mg_2(dobpdc) was saturated with water through vapor diffusion in a sealed vial for *16 hours* (blue trace). Finally, the water-saturated mm-2–Mg_2(dobpdc) was immediately dosed with CS_2 through vapor diffusion in a sealed vial for *10 minutes* (purple trace).

Compiled spectra for Mg_2(dobpdc) are found in Supplementary Figure 83. Mg_2(dobpdc) was pre-activated at 230 °C under flowing N_2 for 8 h. A control spectrum of activated Mg_2(dobpdc) was collected (black trace). Then, activated Mg_2(dobpdc) was dosed with CS_2 through vapor diffusion in a sealed vial for *10 minutes* (red trace). Next, activated Mg_2(dobpdc) was saturated with water through vapor diffusion in a sealed vial for *16 hours* (blue trace). Finally, the water-saturated Mg_2(dobpdc) was immediately dosed with CS_2 through vapor diffusion in a sealed vial for *60 minutes* (purple trace).

Supplementary Tables

Supplementary Table 1 | Crystallographic data

	$Zn_2(dobpdc)(mm\text{-}2\text{–}CS_2)_{1.75}$
Formula	$C_{22.75}H_{27}N_{3.5}O_6S_{3.5}Zn_2$
Temperature (K)	100(2)
Crystal System	Trigonal
Space Group	$P3_121$
a, b, c (Å)	21.6059(7), 21.6059(7), 6.8853(3)
α, β, γ (°)	90, 90, 120
V, (Å^3)	2783.5(2)
Z	3
Radiation, λ (Å)	Synchrotron, 0.7749
2Θ Range for Data Collection (°)	4.110 to 73.222
Completeness to 2Θ	99.8% (2Θ = 55.412°)
Data / Restraints / Parameters	6963 / 69 / 252
Goodness of Fit on F^2	1.080
$R_1{}^a$, $wR_2{}^b$ ($I>2\sigma(I)$)	0.0459, 0.1180
$R_1{}^a$, $wR_2{}^b$ (all data)	0.0537, 0.1221
Largest Diff. Peak and Hole (e Å^{-3})	1.801 and −0.810

[a]$R_1 = \sum||F_o| - |F_c||/\sum|F_o|$. [b]$wR_2 = \{\sum[w(F_o{}^2 - F_c{}^2)^2]/\sum[w(F_o{}^2)^2]\}^{1/2}$.

Supplementary Table 2 | Comparison of calculated structure parameters using different DFT functionals.

	Functional	a (Å)	c (Å)	Zn–N(S) distance (Å)	Zn–Zn distance (Å)
Activated mm-2–Zn_2(dobpdc)	PBE	21.49	6.96	2.19	3.20
	D2	21.18	6.02	2.14	3.37
	vdW-DF2	21.50	6.10	2.21	3.43
	Exp. (XRD)	21.48	6.86	2.14	3.14
CS_2 dosed mm-2–Zn_2(dobpdc)	PBE	21.88	7.05	2.51	3.28
	D2	21.70	6.91	2.46	3.22
	vdW-DF2	21.86	7.07	2.57	3.29
	Exp. (XRD)	21.61	6.89	2.48	3.16
	Functional	a (Å)	c (Å)	Mg–N(S) distance (Å)	Mg–Mg distance (Å)
Activated mm-2–Mg_2(dobpdc)	PBE	21.15	7.02	2.26	3.11
	D2	20.71	6.86	2.21	3.05
	vdW-DF2	20.95	6.85	2.27	3.09
CS_2 dosed mm-2–Mg_2(dobpdc)	PBE	21.80	7.06	2.71	3.12
	D2	21.62	6.91	2.67	3.06
	vdW-DF2	21.76	7.05	2.72	3.12

Supplementary Figures

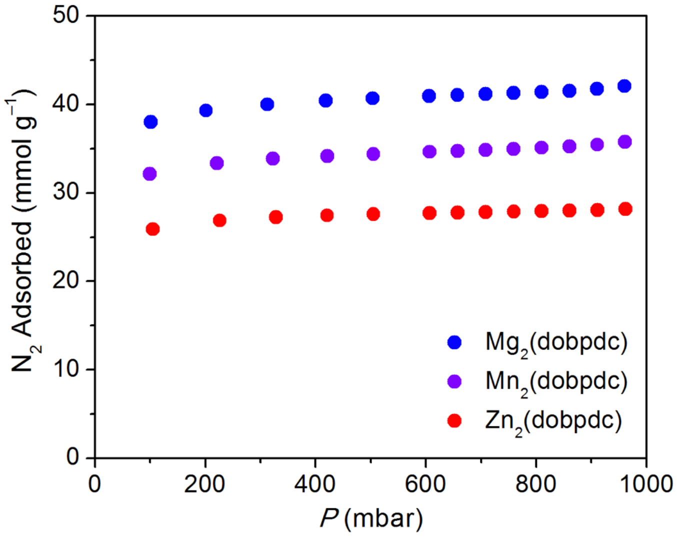

Supplementary Figure 1 | 77 K N_2 adsorption isotherms of activated frameworks. The Langmuir surface areas calculated from this data are indicated in the Method sections.

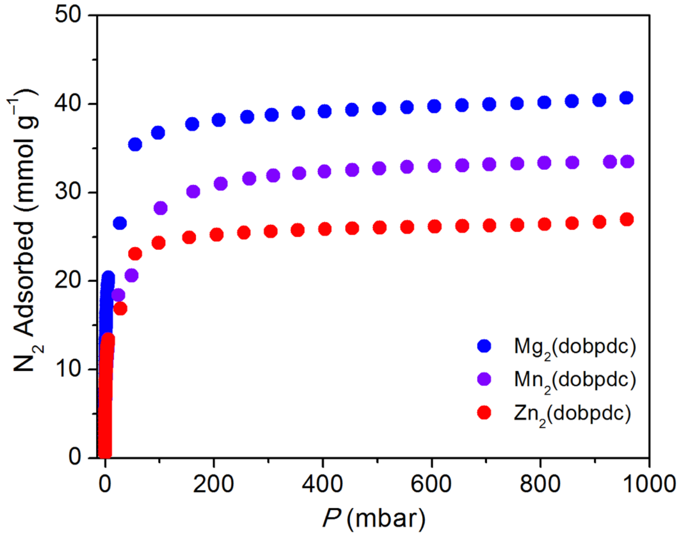

Supplementary Figure 2 | 77 K N_2 adsorption isotherms of activated frameworks.

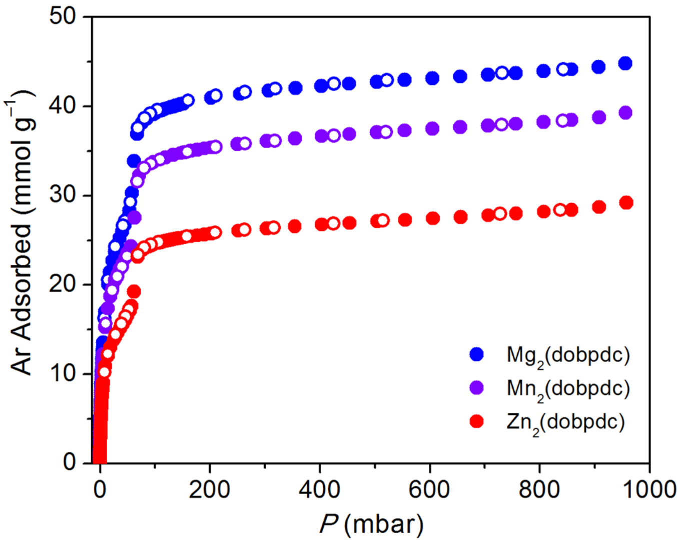

Supplementary Figure 3 | 87 K Ar adsorption isotherms of activated frameworks. Closed circles = adsorption. Open circles = desorption

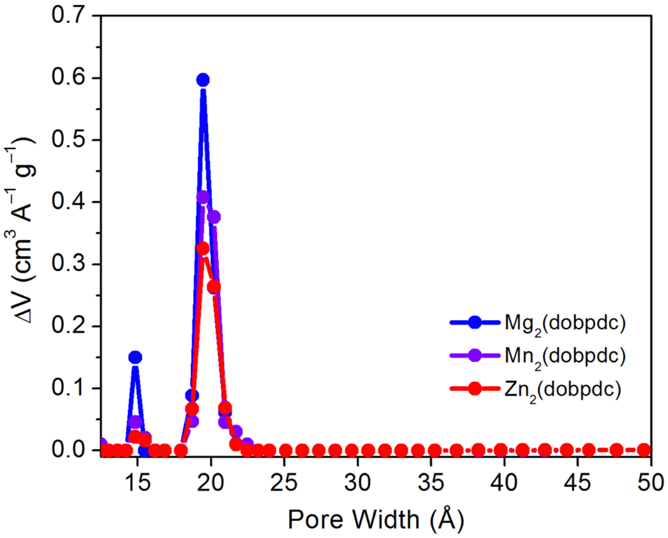

Supplementary Figure 4 | Pore size distribution from 87 K Ar adsorption isotherms of activated frameworks.

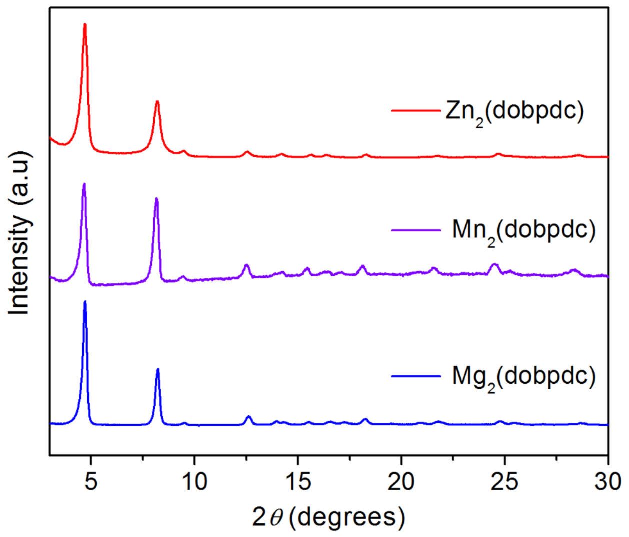

Supplementary Figure 5 | Powder X-ray diffraction patterns of M_2(dobpdc) frameworks in air at 25 °C. CuKα radiation, λ = 1.5418 Å.

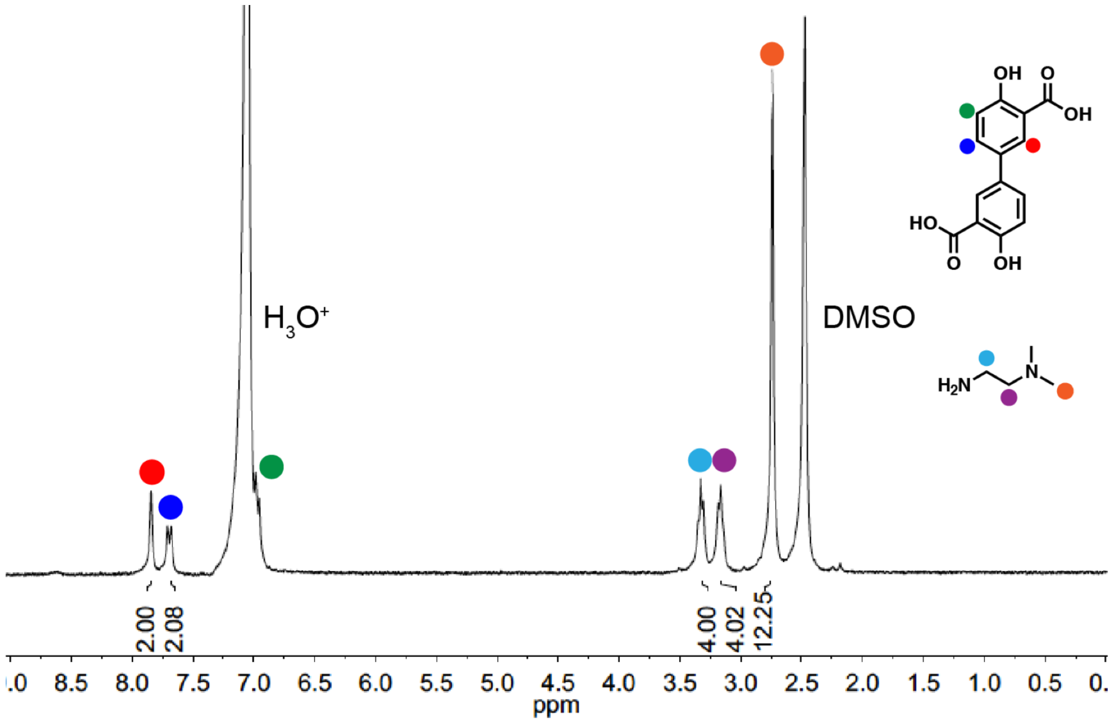

Supplementary Figure 6 | ^{1}H NMR spectrum of digested mm-2–Mg_2(dobpdc). ~10 mg in 0.1 mL 35% DCl in D_2O and 0.6 mL DMSO–d_6. 300 MHz. Assuming a loading of one diamine per Mg^{2+} center, the ratio of diamine to ligand is 2:1.

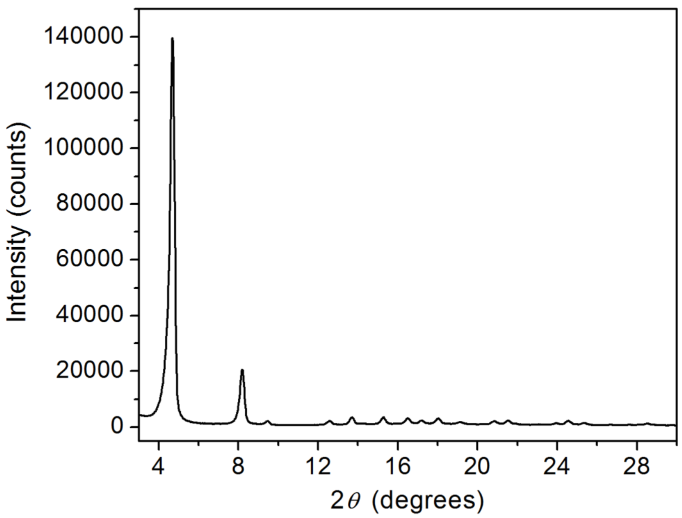

Supplementary Figure 7 | Powder X-ray diffraction pattern of mm-2–Mg_2(dobpdc) in air at 25 °C. CuKα radiation, λ = 1.5418 Å.

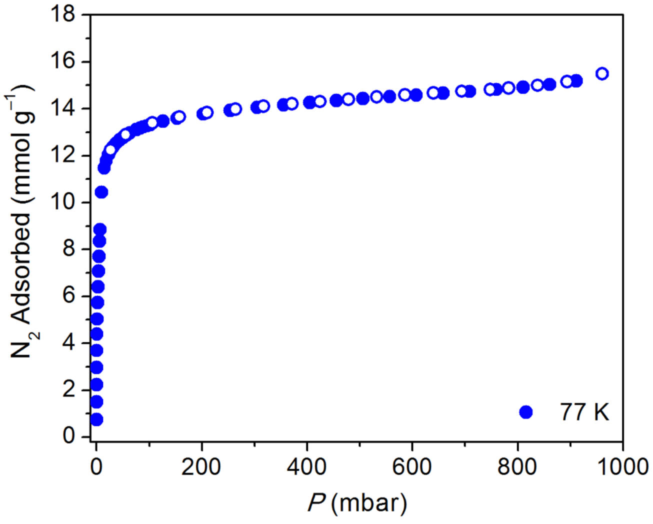

Supplementary Figure 8 | 77 K N_2 adsorption isotherm of mm-2–Mg_2(dobpdc). Activated at 80 °C for 16 h under dynamic vacuum. BET surface area: 1210 m^2 g^{-1}.

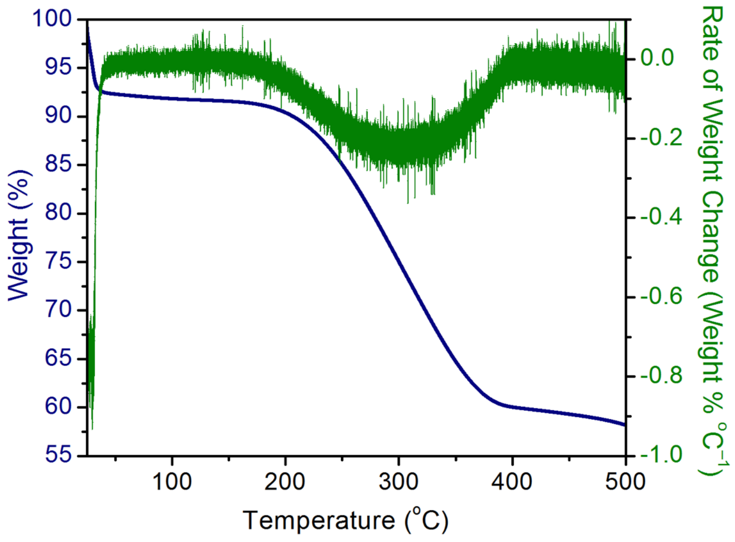

Supplementary Figure 9 | Thermogravimetric analysis decomposition of mm-2–Mg_2(dobpdc). Thermogravimetric analyses were conducted using a TA Instruments Discovery Series TGA with a flow rate of 25 mL min^{-1} for all gases. Masses were uncorrected for buoyancy effects. Heated under flowing N_2 at 1.5 °C min^{-1}. Green line is 1st derivative of weight % versus temperature. The mass loss between 200 and 375 °C corresponds to the volatilization of mm-2 from the framework.

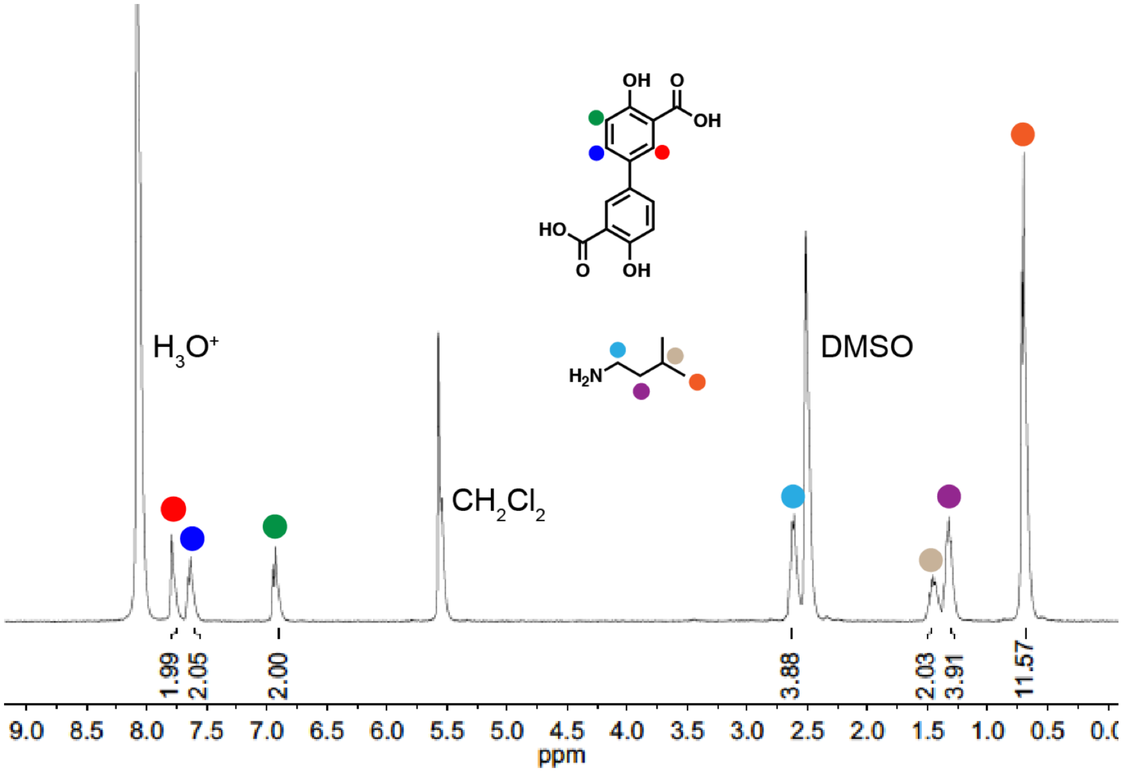

Supplementary Figure 10 | ^{1}H NMR spectrum of digested isopentylamine–Mg_2(dobpdc). ~10 mg in 0.1 mL 35% DCl in D_2O and 0.6 mL DMSO–d_6. 400 MHz. Assuming a loading of one amine per Mg^{2+} center, the ratio of diamine to ligand is 2:1. Spectrum contains residual CH_2Cl_2 from NMR tube cleaning.

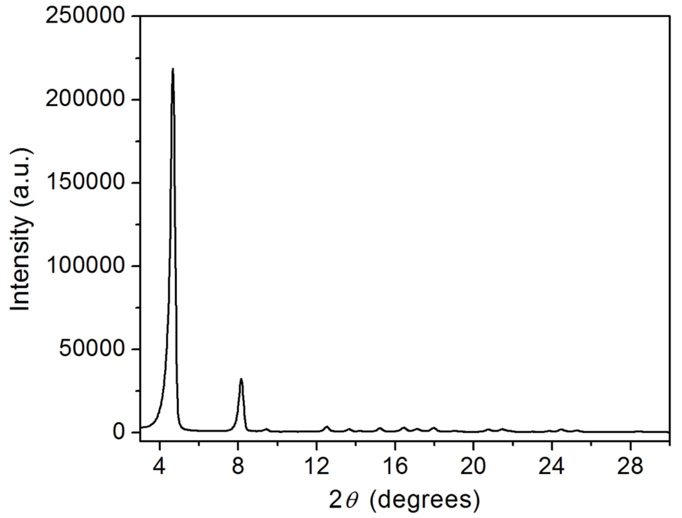

Supplementary Figure 11 | Powder X-ray diffraction pattern of isopentylamine–Mg_2(dobpdc) in air at 25 °C. CuKα radiation, λ = 1.5418 Å.

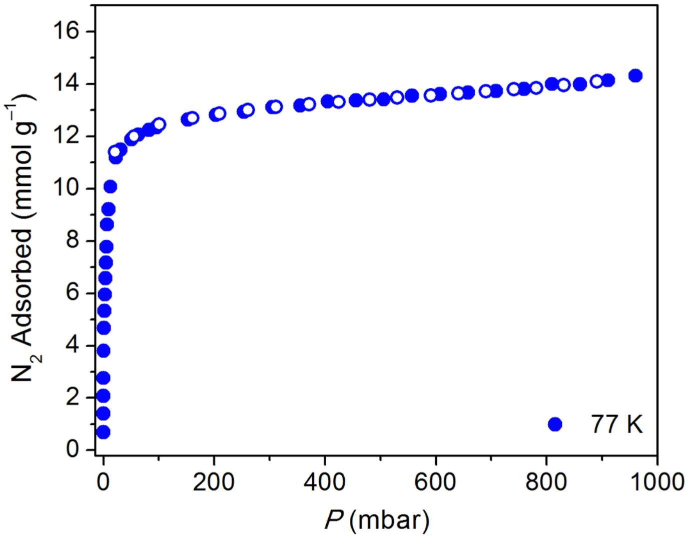

Supplementary Figure 12 | 77 K N_2 adsorption isotherm of isopentylamine–Mg_2(dobpdc). Activated at 80 °C under dynamic vacuum for 16 h. BET surface area: 1130 m^2 g^{-1}.

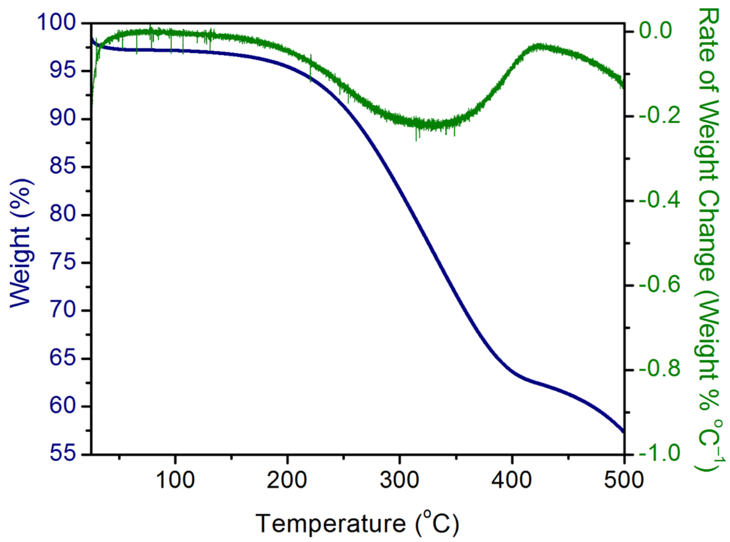

Supplementary Figure 13 | Thermogravimetric analysis decomposition of isopentylamine–Mg_2(dobpdc). Thermogravimetric analyses were conducted using a TA Instruments TGA Q5000 with a flow rate of 25 mL min^{-1} for all gases. Masses were uncorrected for buoyancy effects. Decomposition trace was collected under 100% N_2 with a temperature ramp rate of 1.5 °C min^{-1}. Green line is 1st derivative of weight % versus temperature. The mass loss between 200 and 400 °C corresponds to the volatilization of isopentylamine from the framework.

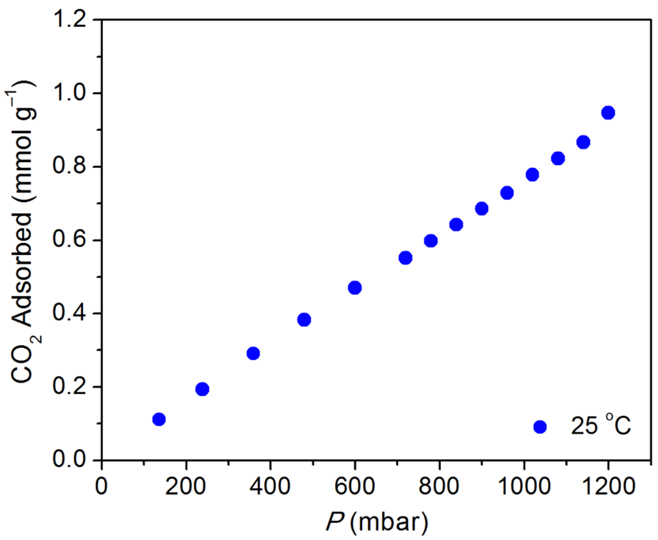

Supplementary Figure 14 | 25 °C CO_2 adsorption isotherm for isopentylamine–Mg_2(dobpdc). Activated at 80 °C under dynamic vacuum for 16 h. In contrast, mm-2–Mg_2(dobpdc) displays step-shaped adsorption of CO_2 in isothermal measurments[2].

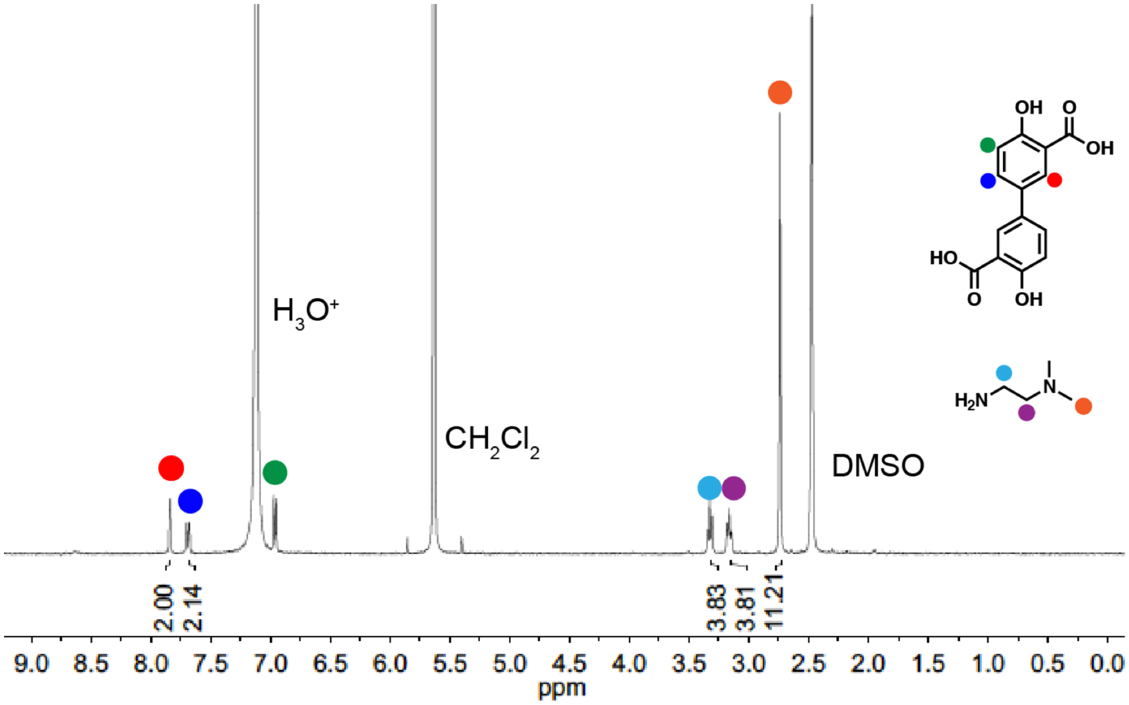

Supplementary Figure 15 | ^{1}H NMR spectrum of digested mm-2–Zn_2(dobpdc). Activated at 100 °C for 2 h under flowing N_2. ~10 mg in 0.1 mL 35% DCl in D_2O and 0.6 mL DMSO–d_6. 400 MHz. ^{1}H NMR spectroscopy digestion confirmed the diamine loading of mm-2–Zn_2(dobpdc) to be ~95%. Assuming a loading of one diamine per Zn^{2+} center, the ratio of diamine to ligand is 2:1. Spectrum contains residual CH_2Cl_2 from NMR tube cleaning. Note: Due to the presence of paramagnetic Mn^{2+} ions in solution, ^{1}H NMR spectra could not be used to reliably quantify diamine loading for Mn_2(dobpdc). Nonetheless, isobaric and isothermal CO_2 adsorption experiments (see below) suggest ~100% loading.

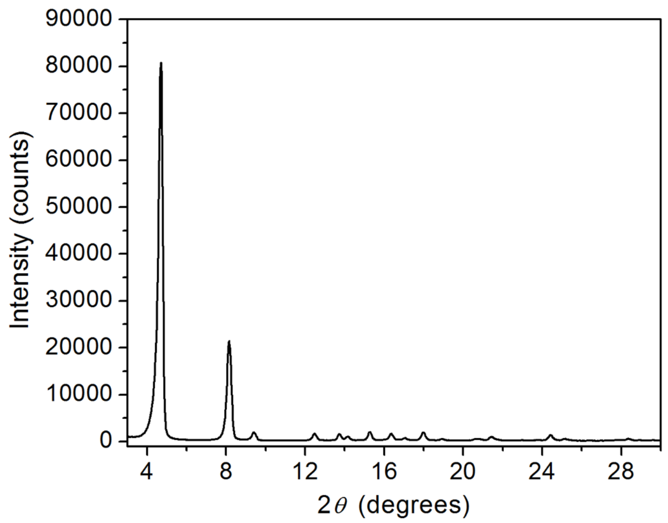

Supplementary Figure 16 | Powder X-ray diffraction pattern of mm-2–Zn_2(dobpdc) in air at 25 °C. CuKα radiation, λ = 1.5418 Å.

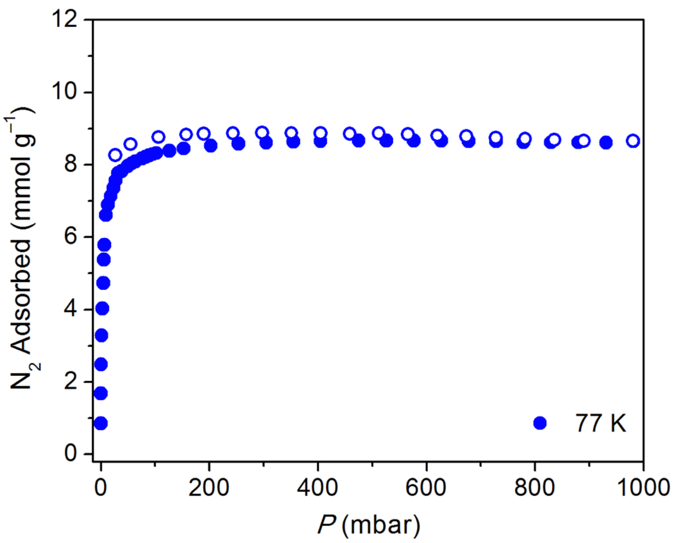

Supplementary Figure 17 | 77 K N_2 adsorption isotherm of mm-2–Zn_2(dobpdc). Activated at 80 °C under dynamic vacuum for 16 h. BET surface area: 760 m^2 g^{-1}.

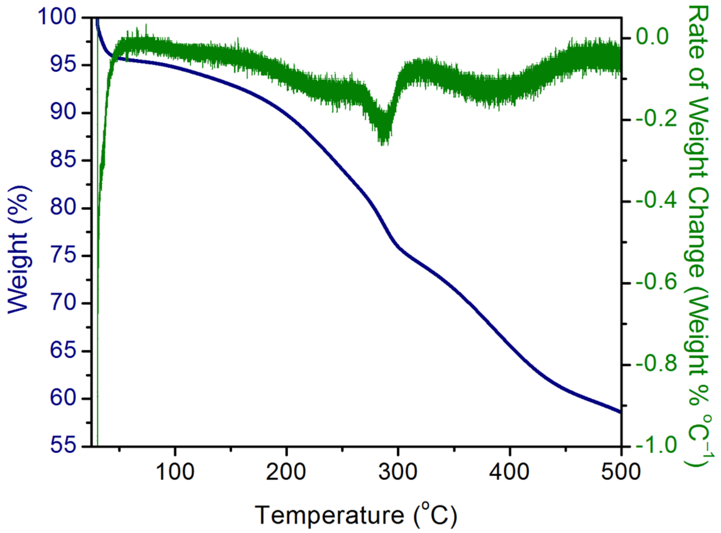

Supplementary Figure 18 | Thermogravimetric analysis decomposition of mm-2–Zn_2(dobpdc). Thermogravimetric analyses were conducted using a TA Instruments Discovery Series TGA with a flow rate of 25 mL min^{-1} for all gases. Masses were uncorrected for buoyancy effects. Decomposition trace was collected under 100% N_2 with a temperature ramp rate of 1.5 °C min^{-1}. Green line is 1st derivative of weight % versus temperature. The mass loss between 150 and 350 °C corresponds to the volatilization of mm-2 from the framework.

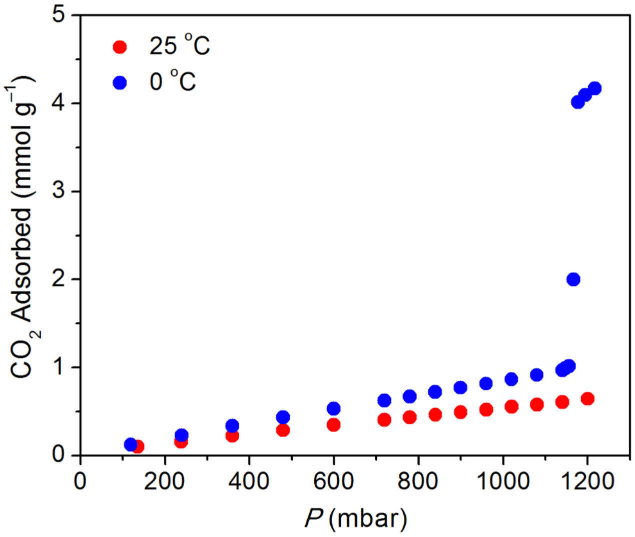

Supplementary Figure 19 | Isothermal CO_2 Adsorption for mm-2–Zn_2(dobpdc) at 25 °C and 0 °C. Activated at 80 °C under dynamic vacuum for 16 h. The chemisorption of one CO_2 per diamine corresponds to 3.44 mmol g^{-1}.

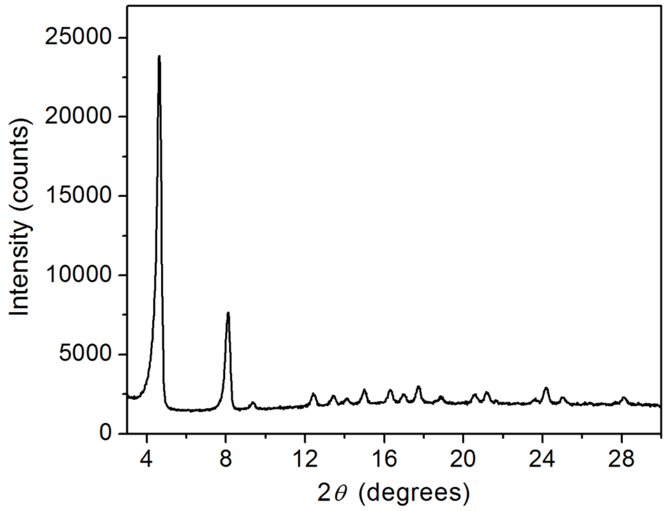

Supplementary Figure 20 | Powder X-ray diffraction pattern of mm-2–Mn_2(dobpdc) in air at 25 °C. CuKα radiation, λ = 1.5418 Å.

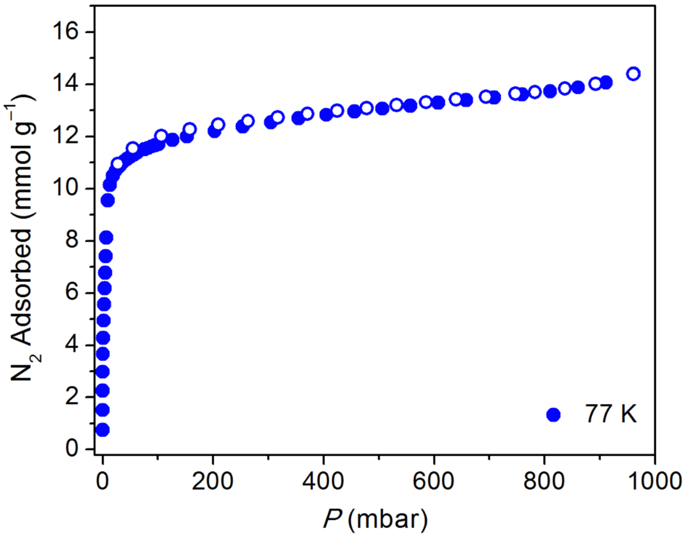

Supplementary Figure 21 | 77 K N_2 adsorption isotherm of activated mm-2–Mn_2(dobpdc). Activated at 80 °C under dynamic vacuum for 16 h. BET surface area: 1070 m^2 g^{-1}.

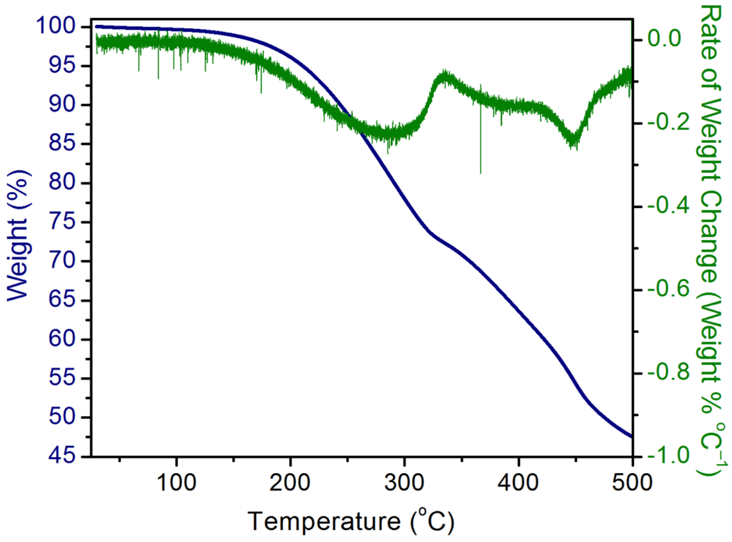

Supplementary Figure 22 | Thermogravimetric analysis decomposition of mm-2–Mn_2(dobpdc). Thermogravimetric analyses were conducted using a TA Instruments TGA Q5000 with a flow rate of 25 mL min^{-1} for all gases. Masses were uncorrected for buoyancy effects. Decomposition trace was collected under 100% N_2 with a temperature ramp rate of 1.5 °C min^{-1}. Green line is 1st derivative of weight % versus temperature. The mass loss between 150 and 350 °C corresponds to the volatilization of mm-2 from the framework.

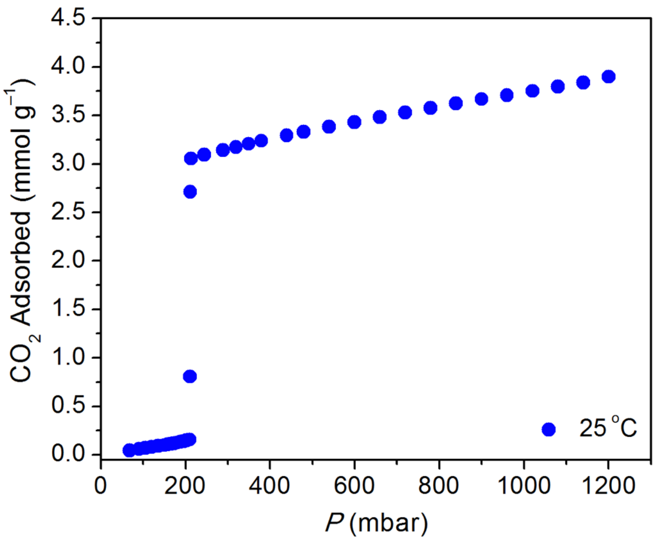

Supplementary Figure 23 | Isothermal CO_2 Adsorption for mm-2–Mn_2(dobpdc) at 25 °C. Activated at 80 °C under dynamic vacuum for 16 h. The adsorption of one CO_2 per diamine corresponds to 3.57 mmol g^{-1}.

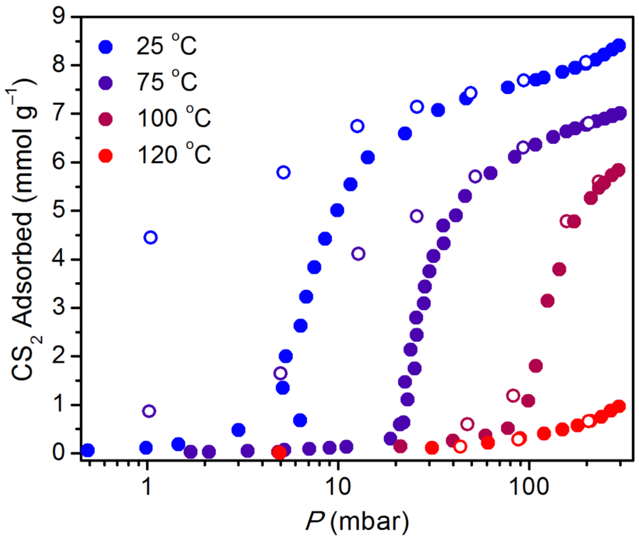

Supplementary Figure 24 | Log plot of CS_2 adsorption isotherms of mm-2–Mg_2(dobpdc). Samples activated at 80 °C under dynamic vacuum. Closed circles = adsorption. Open circles = desorption. Adsorption data plotted here is the same adsorption data shown in Figure 3a.

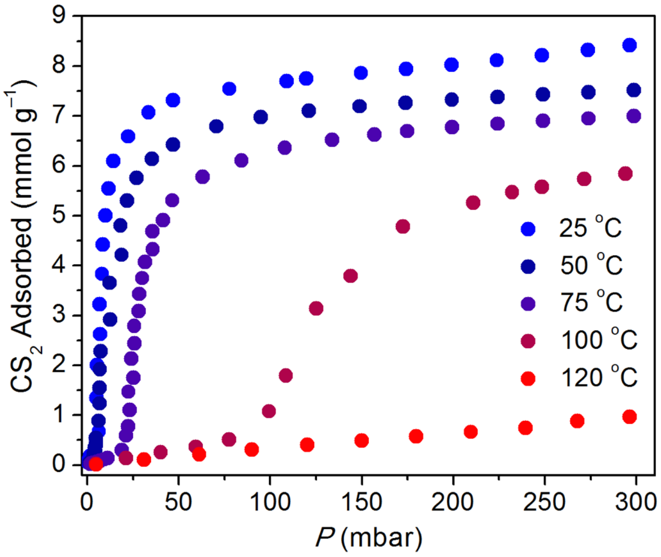

Supplementary Figure 25 | Linear plot of CS_2 adsorption isotherms of mm-2–Mg_2(dobpdc). Samples activated at 80 °C under dynamic vacuum.

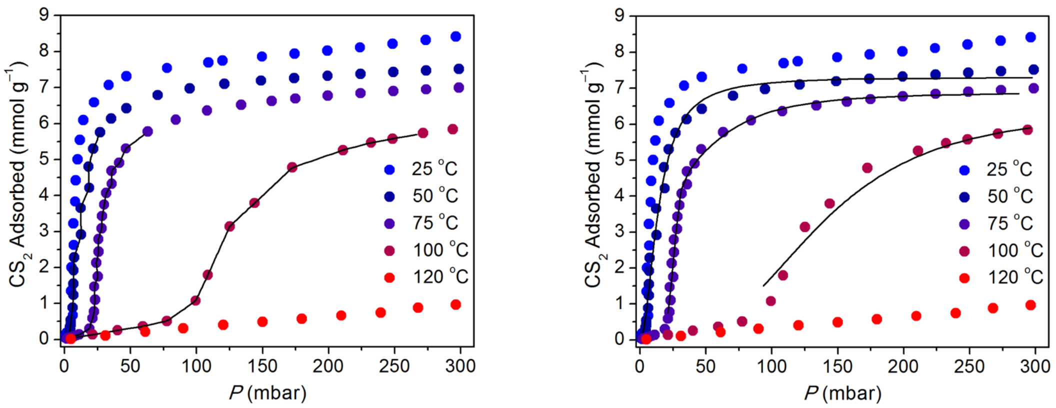

Supplementary Figure 26 | Linear spline interpolation and Fitting of CS_2 adsorption isotherms of mm-2–Mg_2(dobpdc). (Left) Linear interpolation of 50 °C, 75 °C, and 100 °C isotherms performed using a 1st order spline, shown as black lines. At n = 2 mmol g^{-1}, $-\Delta h_{ads}$ = 55 ± 5 kJ mol^{-1} and $-\Delta s_{ads}$ = 129 ± 15 J $(mol·K)^{-1}$. Note: CO_2 adsorption in mm-2–Mg_2(dobpdc) has $-\Delta h_{ads}$ = 52 ± 0.6 kJ mol^{-1} and $-\Delta s_{ads}$ = 146 ± 2 J $(mol·K)^{-1}$ at a loading of 1 mmol g^{-1}[2]. (Right) Mathematical fit of post-step region of 50 °C, 75 °C, and 100 °C isotherms performed using a dual-site Langmuir–Freundlich equation, shown as black lines. At n = 2 mmol g^{-1}, $-\Delta h_{ads}$ = 50 ± 6 kJ mol^{-1}.

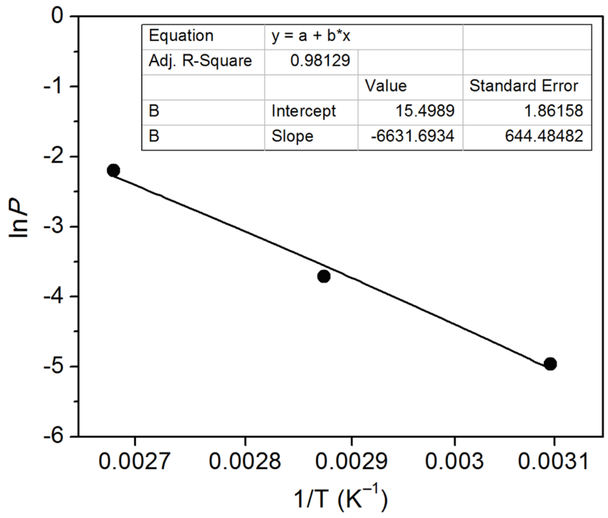

Supplementary Figure 27 | ln P vs. T^{-1} at 2 mmol g^{-1} CS_2 adsorption in mm-2 Mg_2(dobpdc). Representative plot of the pressure–temperature relationship determined from linear interpolation of CS_2 adsorption in mm-2 Mg_2(dobpdc) at 50 °C, 75 °C, and 100 °C. Using the Clausius–Clapeyron relationship $-\Delta h_{ads}$ and $-\Delta s_{ads}$ are determined from the slope and intercept, respectively. Slope = –6630 ± 640 and y-intercept = 15.5 ± 1.9.

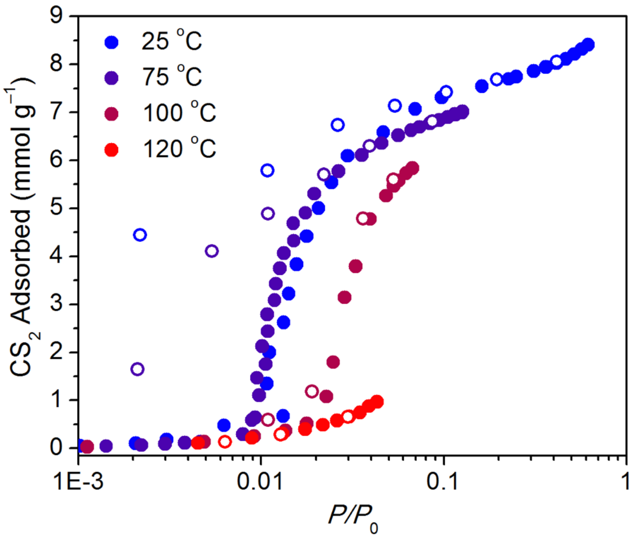

Supplementary Figure 28 | Log plot (*P*/*P*$_0$) of CS_2 adsorption isotherms of mm-2–Mg_2(dobpdc). Samples activated at 80 °C under dynamic vacuum. Closed circles = adsorption. Open circles = desorption. Adsorption data plotted here is the same adsorption data shown in Figure 3a. P_0 determined used the NIST webbook.

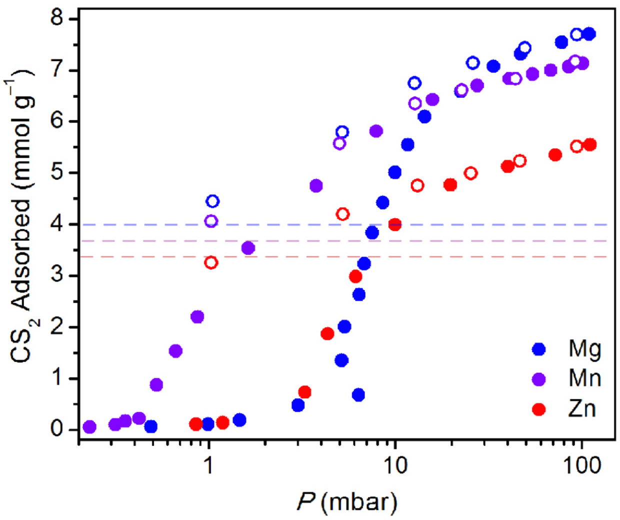

Supplementary Figure 29 | 25 °C CS_2 adsorption isotherms of mm-2–M_2(dobpdc) (M = Mg, Mn, Zn). Samples all activated at 80 °C under dynamic vacuum. Closed circles = adsorption. Open circles = desorption. The x-axis is plotted on a logarithmic scale. Data plotted here is the same data shown in Figure 3c, in which the y-axis units are mmol mmol^{-1}. Dashed lines = capacity assuming the adsorption of one CS_2 per diamine.

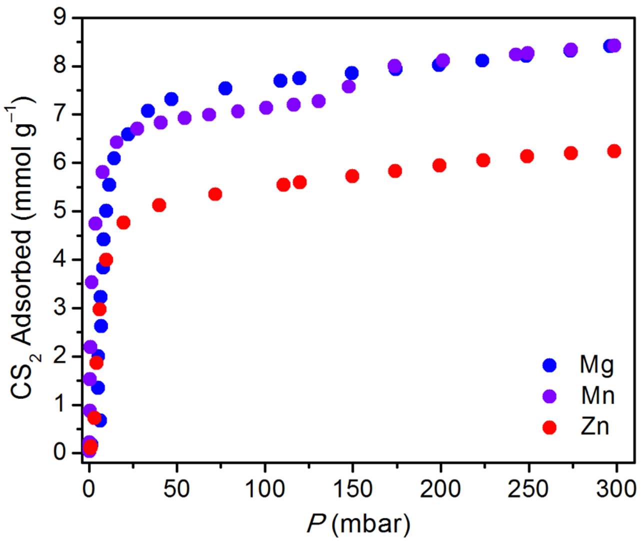

Supplementary Figure 30 | 25 °C CS_2 adsorption isotherms of mm-2–M_2(dobpdc) (M = Mg, Mn, Zn). All activated at 80 °C under dynamic vacuum. The x-axis is plotted on a linear scale. The y-axis units are mmol g^{-1}. Data plotted here is the same data shown in Supplementary Figure 29, without desorption.

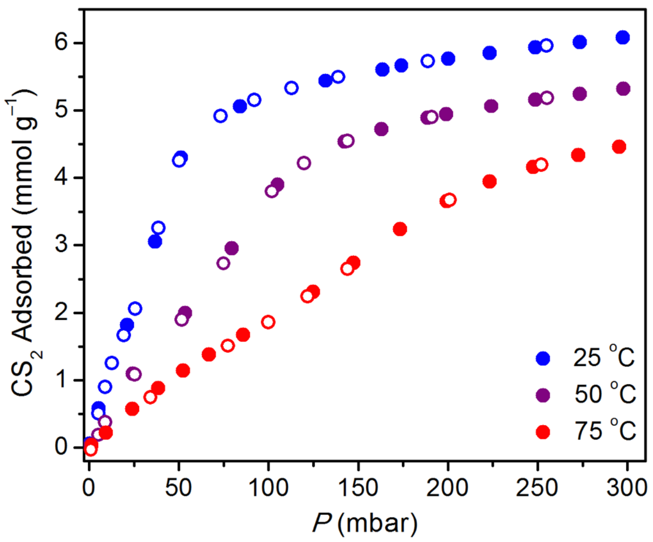

Supplementary Figure 31 | CS_2 adsorption isotherms of isopentylamine–Mg_2(dobpdc). Samples activated at 80 °C under dynamic vacuum. Closed circles = adsorption. Open circles = desorption.

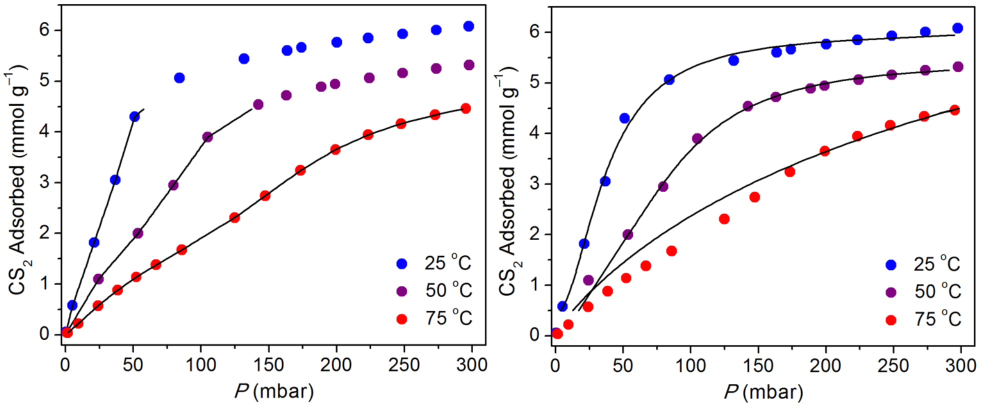

Supplementary Figure 32 | Linear spline interpolation and Fitting of CS_2 adsorption isotherms of isopentylamine–Mg_2(dobpdc). (Left) Linear interpolation performed using a 1st order spline, shown as a black line. At $n = 2$ mmol g^{-1}, $-\Delta h_{ads}$ = 26.0 ± 0.3 kJ mol^{-1} and $-\Delta s_{ads}$ = 56.1 ± 0.9 J (mol·K) $^{-1}$. (Right) Mathematical fit of 25 °C, 50 °C, and 75 °C isotherms performed using a dual-site Langmuir–Freundlich equation, shown as black lines. At $n = 2$ mmol g^{-1}, $-\Delta h_{ads}$ = 21 ± 4 kJ mol^{-1}.

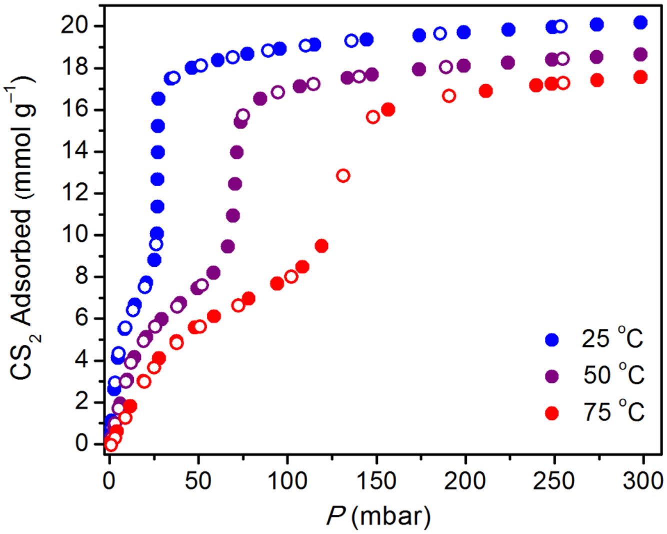

Supplementary Figure 33 | CS_2 adsorption isotherms of Mg_2(dobpdc). Samples activated at 250 °C under dynamic vacuum. Closed circles = adsorption. Open circles = desorption. The adsorption of one CS_2 per Mg^{2+} center corresponds to 6.20 mmol g^{-1}.

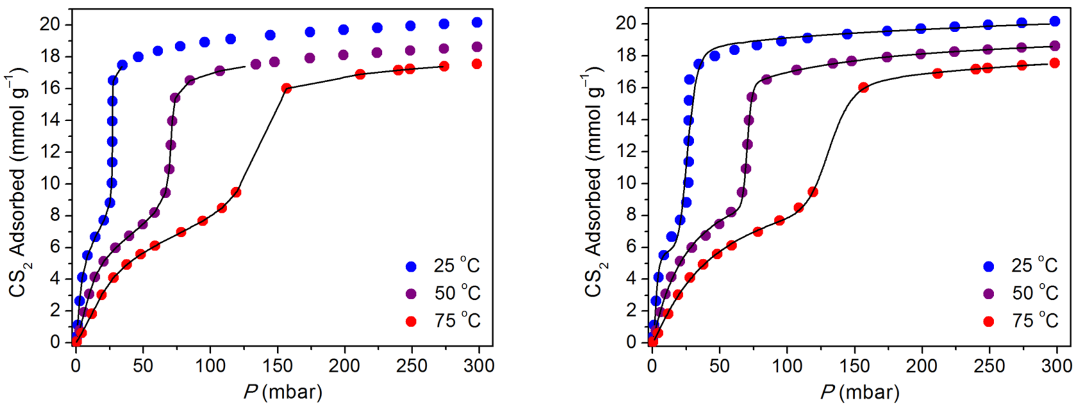

Supplementary Figure 34 | Linear spline interpolation and Fitting of CS_2 adsorption isotherms of Mg_2(dobpdc). (Left) Linear interpolation performed using a 1st order spline, shown as a black line. At n = 2 mmol g^{-1}, $-\Delta h_{ads}$ = 31 ± 2 kJ mol^{-1} and $-\Delta s_{ads}$ = 55 ± 8 J (mol·K)$^{-1}$. At n = 12 mmol g^{-1}, $-\Delta h_{ads}$ = 27 ± 2 kJ mol^{-1} and $-\Delta s_{ads}$ = 63 ± 6 J (mol·K)$^{-1}$. (Right) Mathematical fit of 25 °C, 50 °C, and 75 °C isotherms performed using a triple-site Langmuir–Freundlich equation, shown as black lines. At n = 2 mmol g^{-1}, $-\Delta h_{ads}$ = 28.3 ± 0.8 kJ mol^{-1}. At n = 12 mmol g^{-1}, $-\Delta h_{ads}$ = 28 ± 2 kJ mol^{-1}.

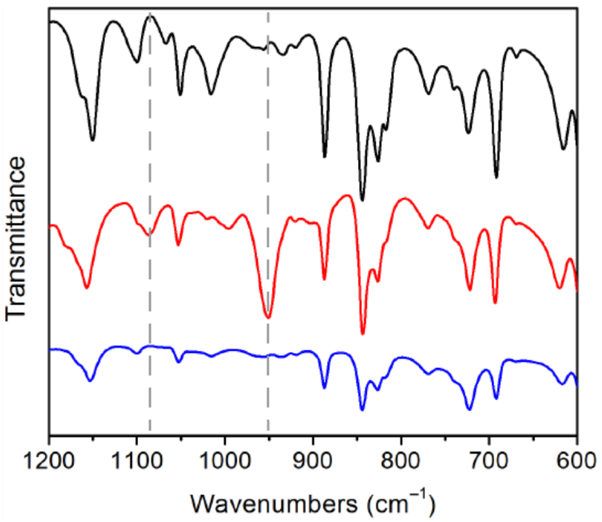

Supplementary Figure 35 | Transmittance mode FT–IR Spectra of mm-2–Mg_2(dobpdc). Black line = activated, red line = CS_2 dosed, and blue line = reactivated at 100 °C under dynamic vacuum (~0.13 mbar) for 15 min. Dashed lines highlight bands at 1085–1089 and 953–955 cm^{-1} arising from C–S vibrations in the CS_2-dosed sample (Supplementary Figures 56–59).

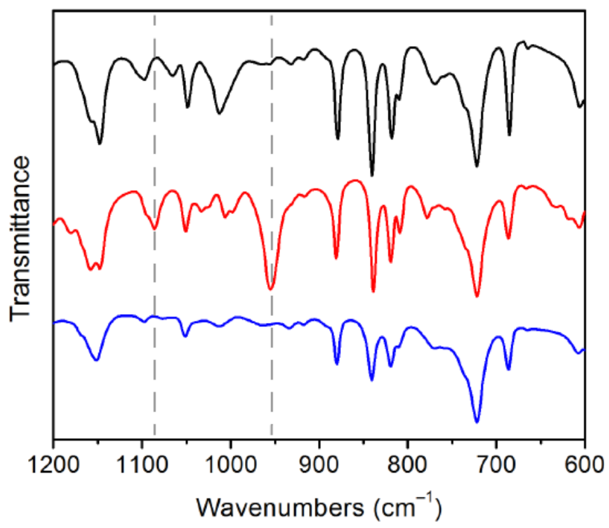

Supplementary Figure 36 | Transmittance mode FT–IR Spectra of mm-2–Mn_2(dobpdc). Black line = activated, red line = CS_2 dosed, and blue line = reactivated at 100 °C under dynamic vacuum (~0.13 mbar) for 2 h. Dashed lines highlight bands at 1085–1089 and 953–955 cm^{-1} arising from C–S vibrations in the CS_2-dosed sample (Supplementary Figures 60–63).

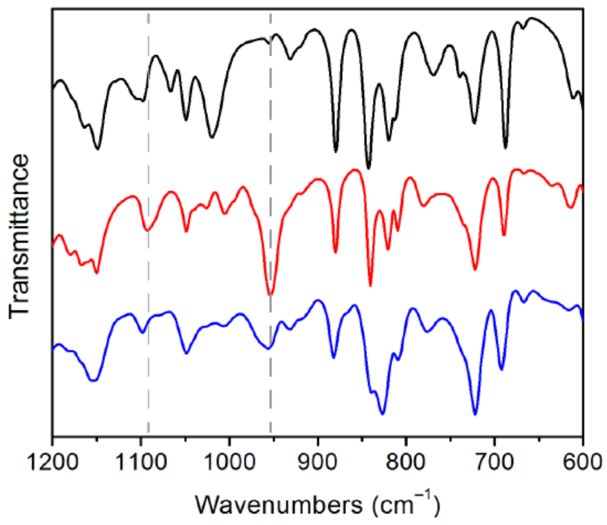

Supplementary Figure 37 | Transmittance mode FT–IR Spectra of mm-2–Zn_2(dobpdc). Black line = activated, red line = CS_2 dosed, and blue line = reactivated at 150 °C under dynamic vacuum (~0.13 mbar) for 2 h. Dashed lines highlight bands at 1085–1089 and 953–955 cm^{-1} arising from C–S vibrations in the CS_2-dosed sample (Supplementary Figures 64–67).

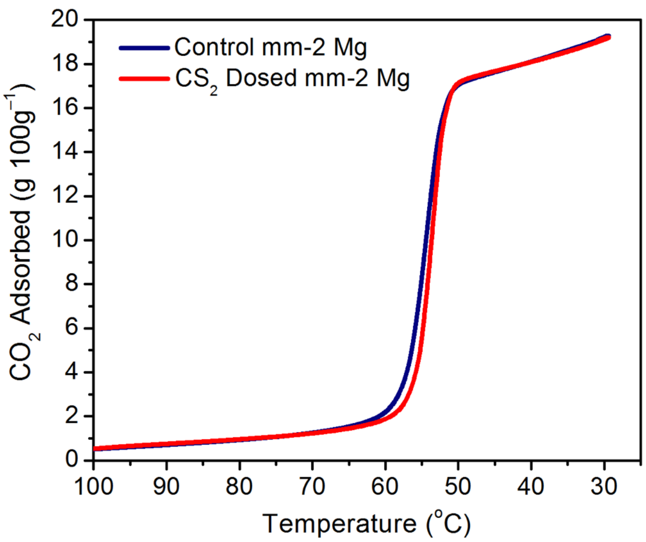

Supplementary Figure 38 | Thermogravimetric analysis: CO_2 isobars of mm-2–Mg_2(dobpdc). CS_2-dosed sample was re-activated under flowing N_2 at 120 °C for 30 min.

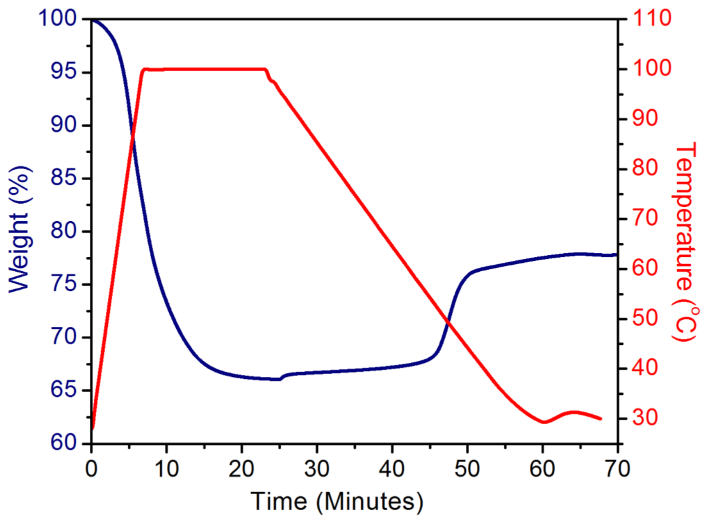

Supplementary Figure 39 | Thermogravimetric analysis: heating under N_2 at 100 °C and cooling under CO_2 for CS_2-dosed mm-2–Mg_2(dobpdc). A CS_2-dosed sample was heated under N_2 to 100 °C and held until the mass equilibrated, upon which the gas flow was switched to CO_2 (at time ≈ 25 min) and the sample was cooled to 30 °C.

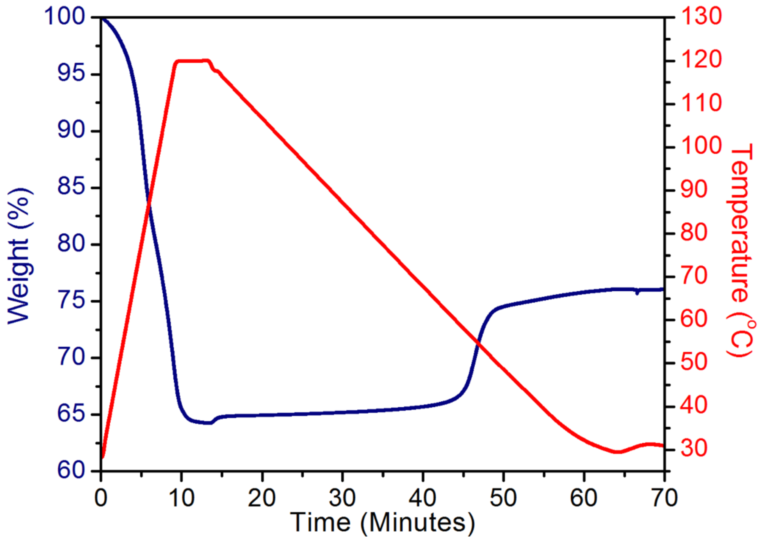

Supplementary Figure 40 | Thermogravimetric analysis: heating under N_2 at 120 °C and cooling under CO_2 for CS_2-dosed mm-2–Mg_2(dobpdc). A CS_2-dosed sample was heated under N_2 to 120 °C and held until the mass equilibrated, upon which the gas flow was switched to CO_2 (at time ≈ 15 min) and the sample was cooled to 30 °C.

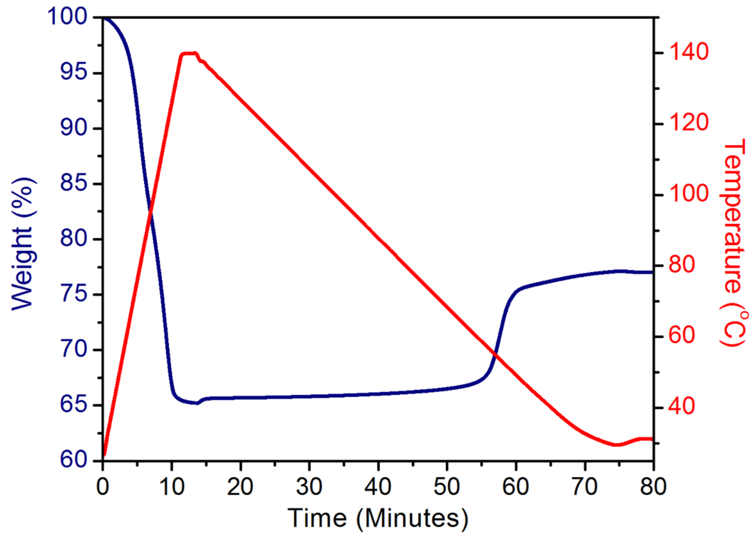

Supplementary Figure 41 | Thermogravimetric analysis: heating under N_2 at 140 °C and cooling under CO_2 for CS_2-dosed mm-2–Mg_2(dobpdc). A CS_2-dosed sample was heated under N_2 to 140 °C and held until the mass equilibrated, upon which the gas flow was switched to CO_2 (at time ≈ 15 min) and the sample was cooled to 30 °C.

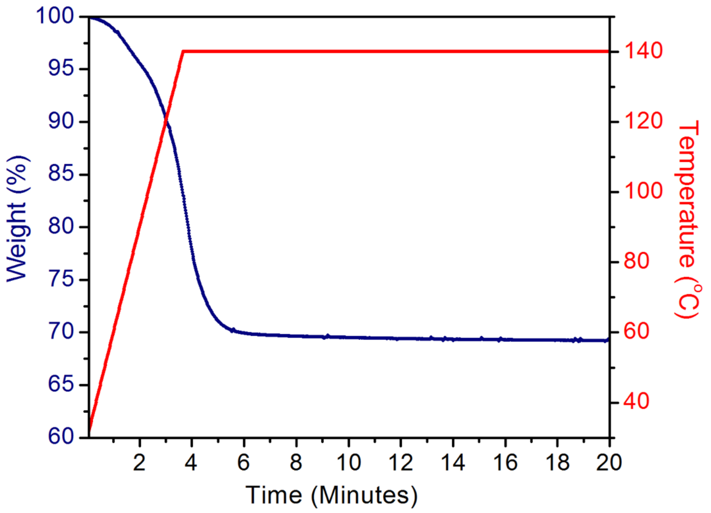

Supplementary Figure 42 | Temperature and weight % versus time for TGA–MS of CS_2-dosed mm-2–Mg_2(dobpdc). TGA–MS performed using a Perkin Elmer Pyris 1 TGA paired with a Hiden Analytical HPR20 Sampling System. In order to remove physisorbed CS_2 before the measurement, the CS_2-dosed sample was exposed to dynamic vacuum (~0.13 mbar) for 16 h at 25 °C. The sample was heated under N_2 flow to 140 °C.

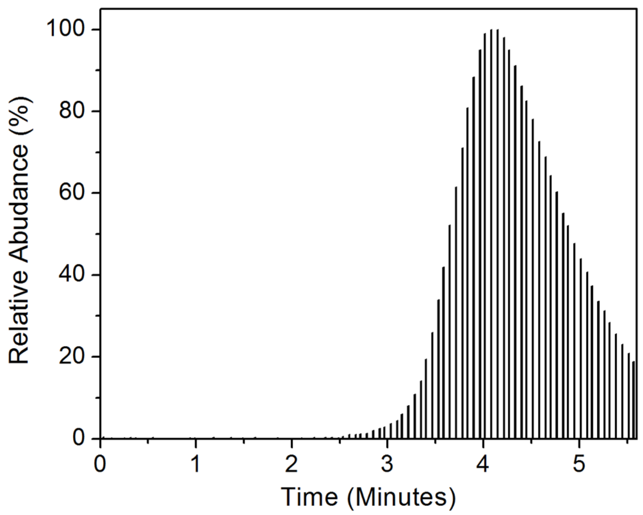

Supplementary Figure 43 | Relative abundance of 76 m/z (CS_2) versus time for TGA–MS of CS_2-dosed mm-2–Mg_2(dobpdc). Taken during experiment shown in Supplementary Figure 42. TGA–MS performed using a Perkin Elmer Pyris 1 TGA paired with a Hiden Analytical HPR20 Sampling System. In order to remove physisorbed CS_2 before the measurement, the CS_2-dosed sample was exposed to dynamic vacuum (~0.13 mbar) for 16 h at 25 °C. The sample was heated under N_2 flow to 140 °C.

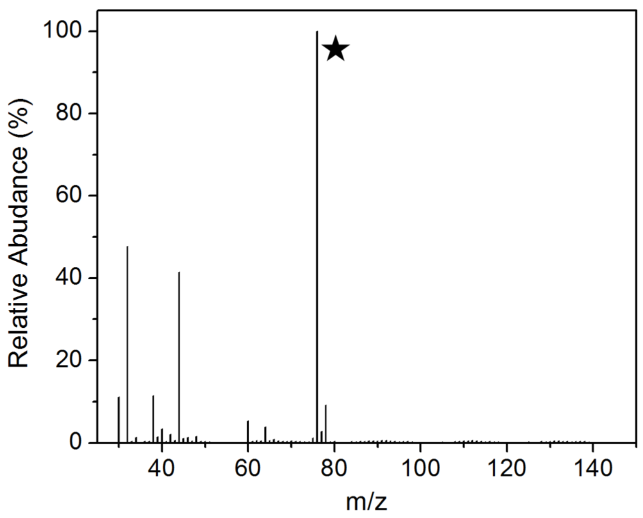

Supplementary Figure 44 | Snapshot of m/z at time = 250 seconds for TGA–MS of CS_2-dosed mm-2–Mg_2(dobpdc). Taken during experiment shown in Supplementary Figure 42. TGA–MS performed using a Perkin Elmer Pyris 1 TGA paired with a Hiden Analytical HPR20 Sampling System. CS_2 is seen at 76 m/z (starred). In order to remove physisorbed CS_2 before the measurement, the CS_2-dosed sample was exposed to dynamic vacuum (~0.13 mbar) for 16 h at 25 °C. The sample was heated under N_2 flow to 140 °C.

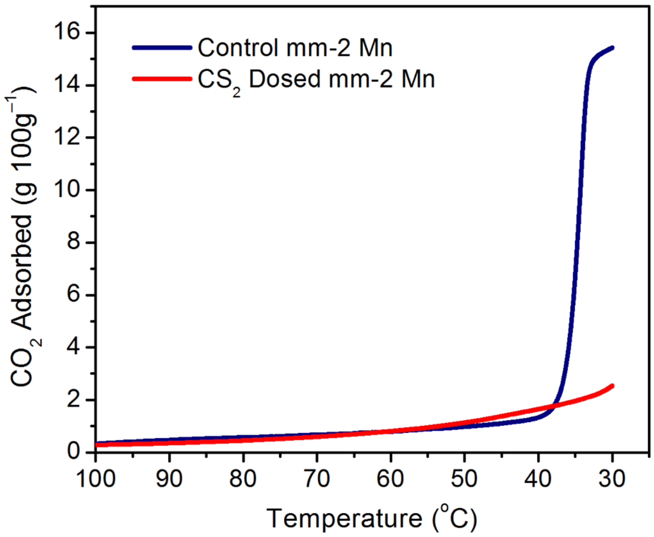

Supplementary Figure 45 | Thermogravimetric analysis: CO_2 isobars of mm-2–Mn_2(dobpdc). CS_2-dosed sample was re-activated under flowing N_2 at 120 °C for 30 min.

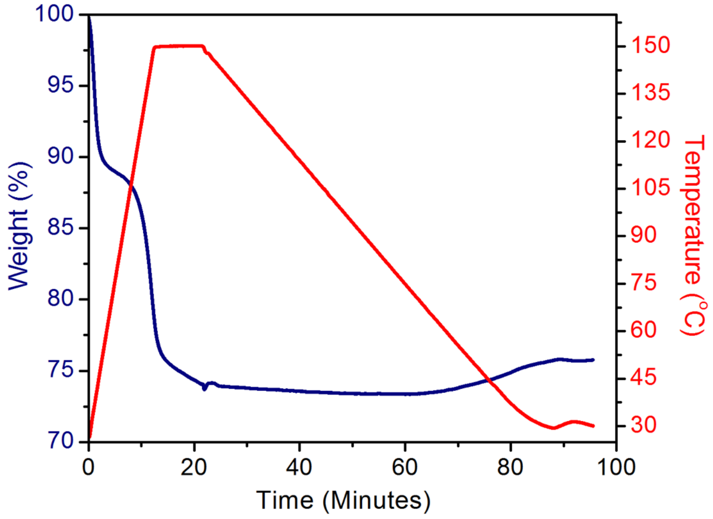

Supplementary Figure 46 | Thermogravimetric analysis: heating under N_2 at 150 °C and cooling under CO_2 for CS_2-dosed mm-2–Mn_2(dobpdc). A CS_2-dosed sample was heated under N_2 to 150 °C and held until the mass equilibrated, upon which the gas flow was switched to CO_2 (at time ≈ 25 min) and the sample was cooled to 30 °C. Note: for this sample dosing was achieved by exposing mm-2–Mn_2(dobpdc) to CS_2 vapor in a 20 mL scintillation vial for 30 min. As vacuum desorption at 25 °C was not performed before analysis some weight loss is initially observed at low temperature under flowing N_2 owing to weakly physisorbed CS_2.

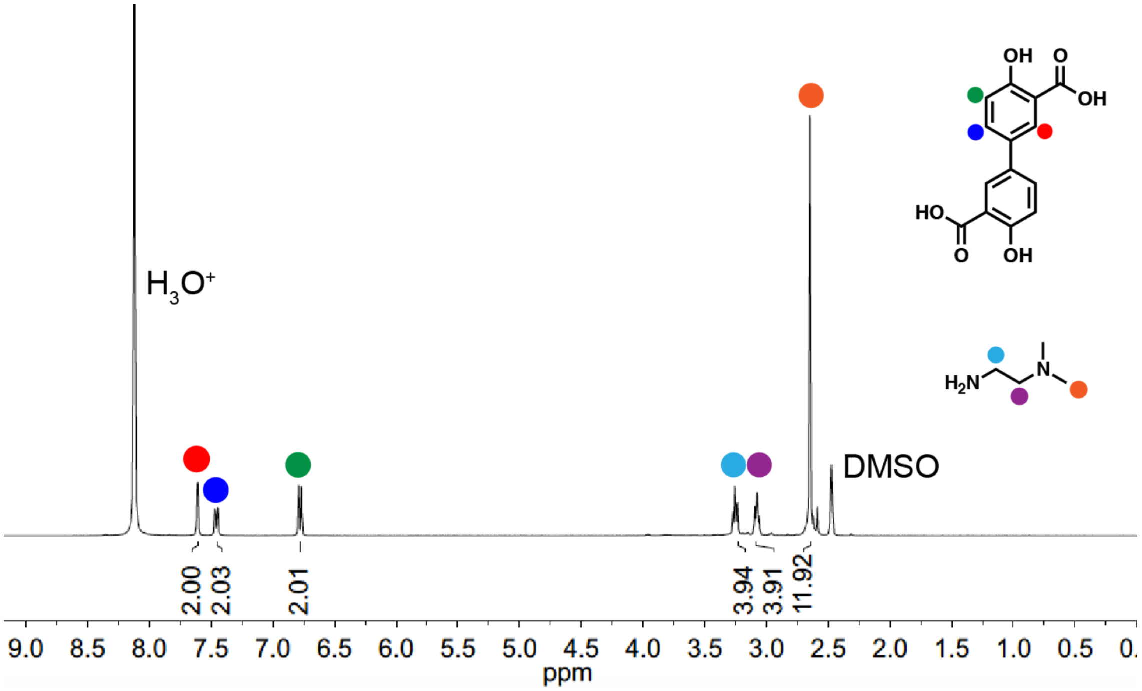

Supplementary Figure 47 | ^{1}H NMR spectrum of digested mm-2–Mg_2(dobpdc) after 10 cycles of CS_2 adsorption–desorption. ~10 mg in 0.1 mL 35% DCl in D_2O and 0.6 mL DMSO–*d*$_6$. 400 MHz. Assuming a loading of one diamine per Mg^{2+} center, the ratio of diamine to ligand is 2:1.

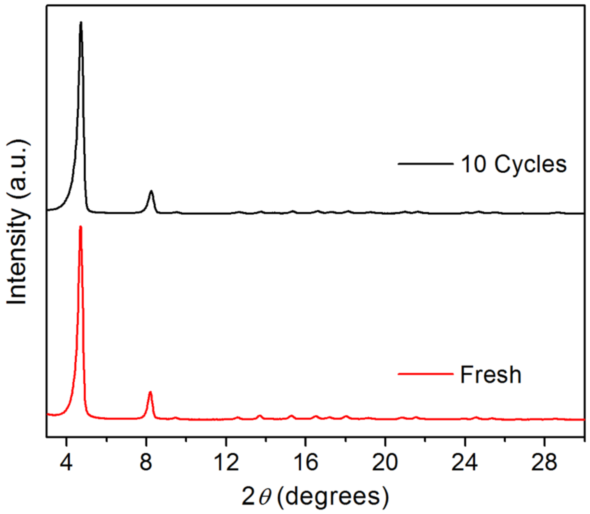

Supplementary Figure 48 | Powder X-ray diffraction pattern of mm-2–Mg_2(dobpdc) in air at 25 °C before and after 10 CS_2 adsorption–desorption cycles. Black line = mm-2–Mg_2(dobpdc) after 10 cycles of CS_2 adsorption–desorption. Red line = fresh activated mm-2–Mg_2(dobpdc).

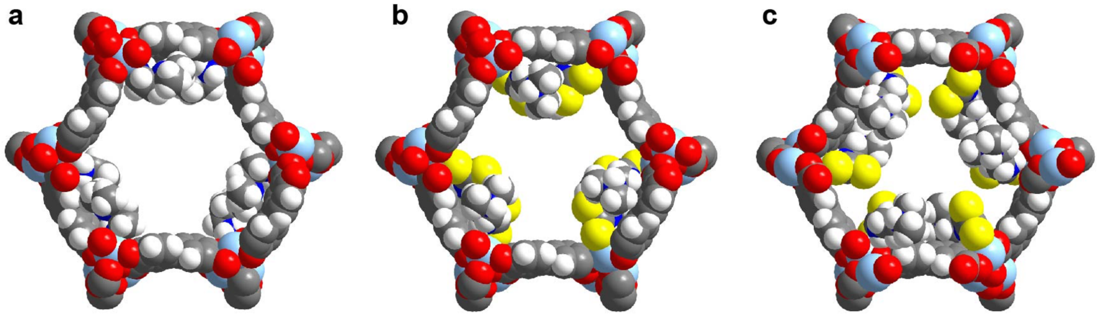

Supplementary Figure 49 | Space-filling representations of single-crystal X-ray diffraction structures. **a**, A portion of the structure of mm-2−Zn_2(dobpdc) viewed in the *ab* plane. **b**, A portion of the structure of one conformation of CS_2-inserted mm-2−Zn_2(dobpdc) viewed in the *ab* plane. **c**, A portion of the structure of the second conformation of CS_2-inserted mm-2−Zn_2(dobpdc) viewed in the *ab* plane. All structures were collected at 100 K. Structures were refined as inversion twins in either space group $P3_121$ or $P3_221$. For clarity, all images are shown in the $P3_221$ space group. Light blue, blue, red, gray, yellow, and white spheres represent Zn, N, O, C, S, and H atoms, respectively.

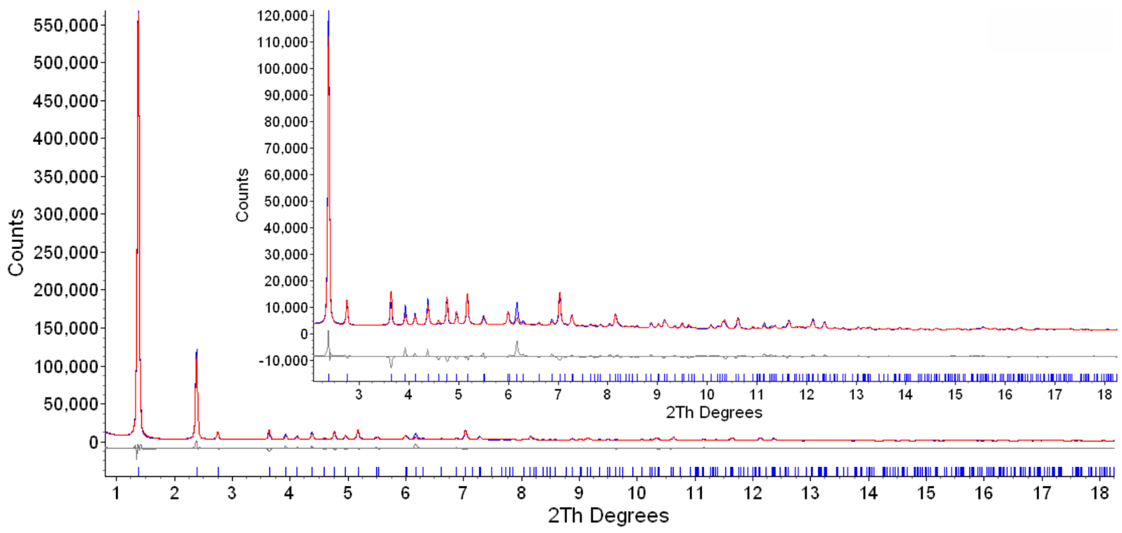

Supplementary Figure 50 | Rietveld refinement plot of CS_2-inserted mm-2–Mn_2(dobpdc) collected at 100 K at a wavelength of 0.45241 Å. Blue and red lines represent the observed and calculated diffraction patterns, respectively. The gray line represents the difference between observed and calculated patterns, and the blue tick marks indicate calculated Bragg peak positions. The inset shows the high angle region at a magnified scale.

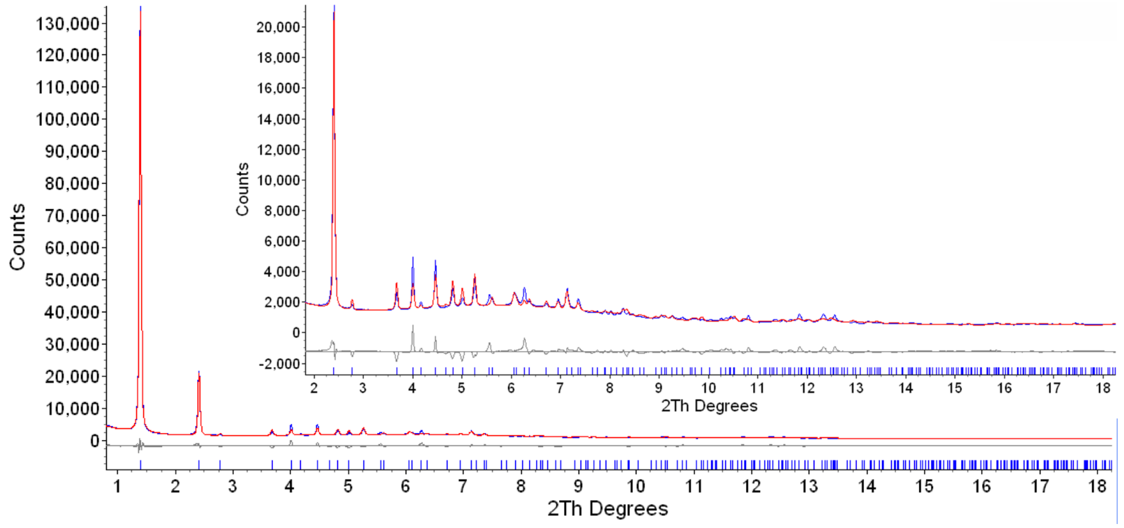

Supplementary Figure 51 | Rietveld refinement plot of CS_2-inserted mm-2–Mg_2(dobpdc) collected at 100 K at a wavelength of 0.45241 Å. Blue and red lines represent the observed and calculated diffraction patterns, respectively. The gray line represents the difference between observed and calculated patterns, and the blue tick marks indicate calculated Bragg peak positions. The inset shows the high angle region at a magnified scale.

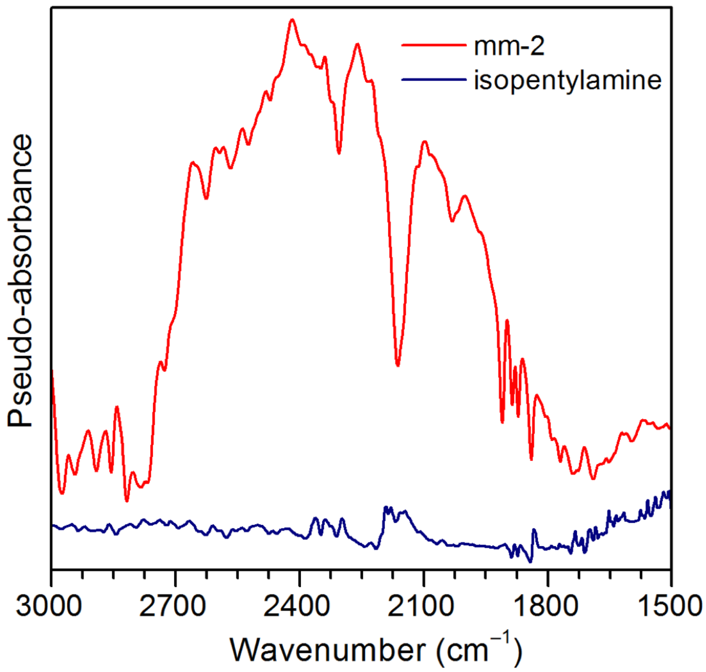

Supplementary Figure 52 | *in-situ* DRIFT spectra upon exposure of activated mm-2−Mg_2(dobpdc) (red) and isopentylamine–Mg_2(dobpdc) (blue) to the vapor pressure of CS_2 at 25 °C. Effects from physisorbed CS_2 on the framework have been subtracted as the baseline to generate difference plots. The broad feature from ~2800 to 1700 cm^{-1} is typical of hydrogen-bonded ammonium species[10].

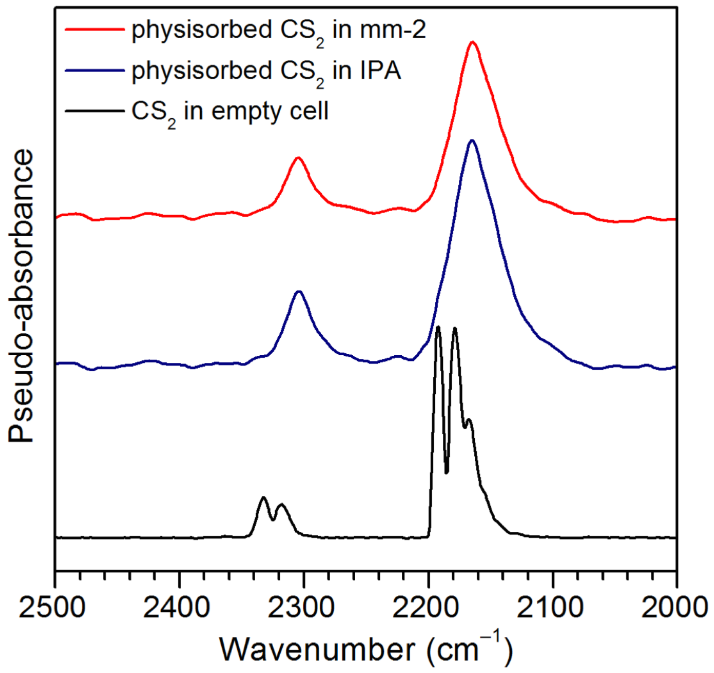

Supplementary Figure 53 | *in-situ* DRIFT spectra upon exposure of mm-2−Mg_2(dobpdc) (red), isopentylamine–Mg_2(dobpdc) (blue), and the empty cell (black) to the vapor pressure of CS_2 at 25 °C. Spectra were collected immediately upon exposure of the sample space to CS_2 in order to observe effects of physisorption in isolation. Spectra of the activated frameworks and evacuated cell have been used as baselines to generate difference plots. Features at 2302 and 2164 cm^{-1} observed in the framework upon CS_2 dosing are distinct from those arising from CS_2 observed in the empty cell and similar to liquid CS_2[11], suggesting condensation within the pores.

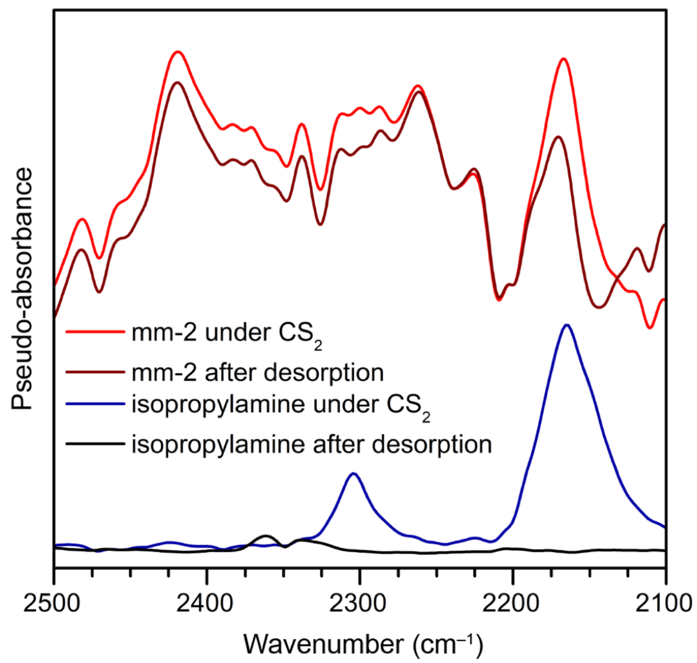

Supplementary Figure 54 | *in-situ* DRIFT spectra of mm-2−Mg_2(dobpdc) after exposure to CS_2 (red), followed by evacuation until spectra stabilized (dark red), and isopentylamine–Mg_2(dobpdc) after exposure to CS_2 (blue), followed by evacuation until spectra stabilized (black). Spectra of the activated frameworks have been used as baselines to generate difference plots, and scaled for clarity. In both materials, peaks attributed to condensed CS_2 disappeared upon evacuation. In mm-2−Mg_2(dobpdc), the feature at 2302 cm^{-1} is obscured by the broad ammonium feature formed upon chemisorption; however, the disappearance of the partially obscured feature at 2164 cm^{-1} is observed.

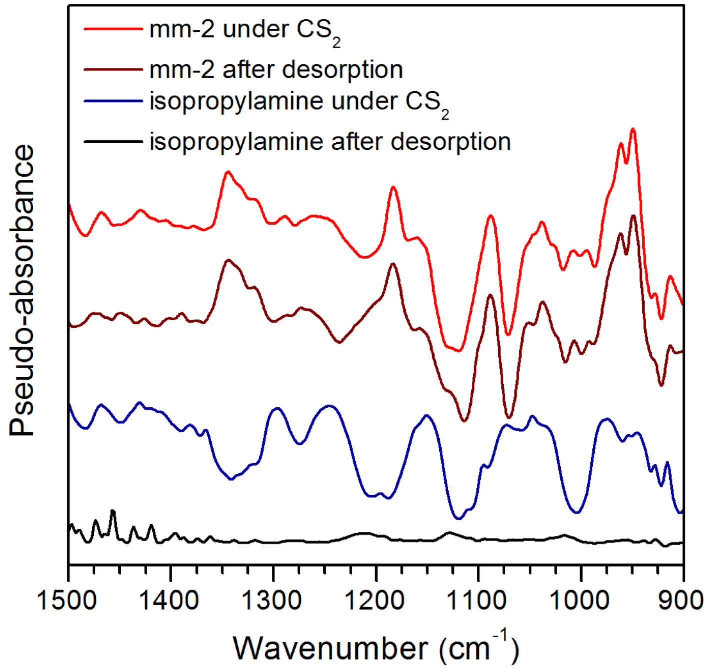

Supplementary Figure 55 | *in-situ* DRIFT spectra of mm-2−Mg_2(dobpdc) after exposure to CS_2 (red), followed by evacuation until spectra stabilized (dark red), and isopentylamine–Mg_2(dobpdc) after exposure to CS_2 (blue), followed by evacuation until spectra stabilized (black). Spectra of the activated frameworks have been used as baselines to generate difference plots. Whereas the spectrum of isopentylamine–Mg_2(dobpdc) reverts back to that of the activated framework upon evacuation after CS_2 dosing, in mm-2−Mg_2(dobpdc) features at 1343, 1318, 961, and 949 cm^{-1} resulting from chemisorption remain.

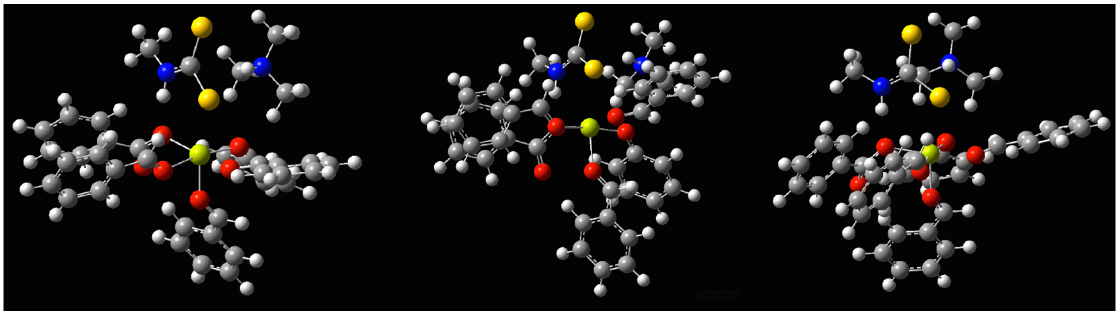

Supplementary Figure 56 | Geometrically optimized truncated single-site model of CS_2-dosed mm-2–Mg_2(dobpdc). All three images are of the same model from different angles. Grey, white, yellow, blue, red, and green spheres represent C, H, S, N, O, and Mg atoms, respectively.

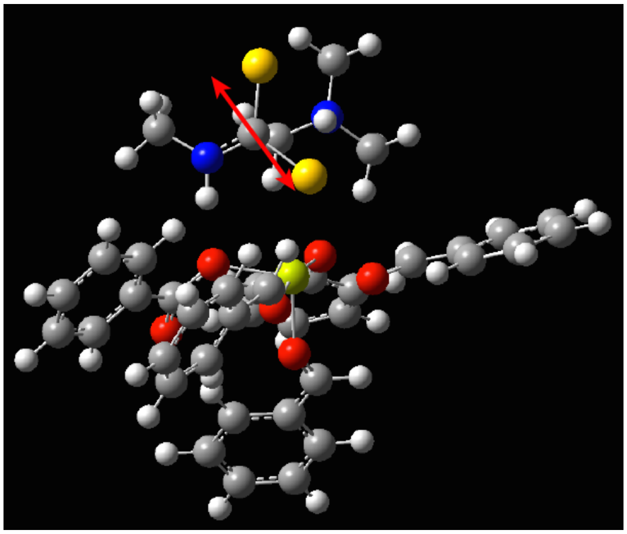

Supplementary Figure 57 | Vibration calculation of geometrically optimized truncated single-site model of CS_2-dosed mm-2–Mg_2(dobpdc). The red arrow on the carbon of the dithiocarbamate highlights a vibration calculated to occur at 944 cm^{-1}. Grey, white, yellow, blue, red, and green spheres represent C, H, S, N, O, and Mg atoms, respectively.

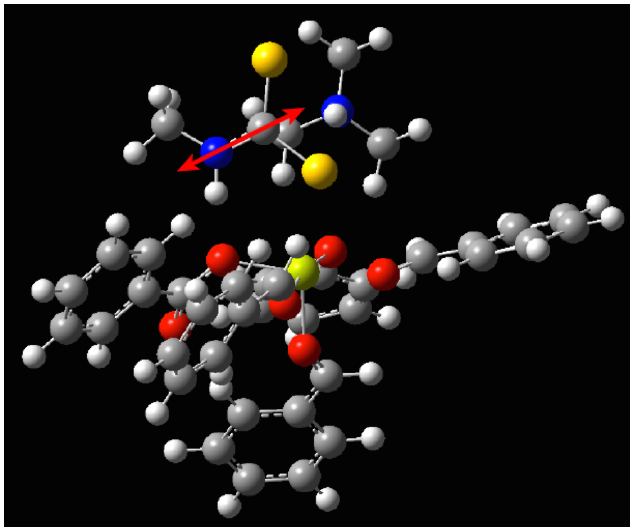

Supplementary Figure 58 | Vibration calculation of geometrically optimized truncated single-site model of CS_2-dosed mm-2–Mg_2(dobpdc). The red arrow on the carbon and nitrogen of the dithiocarbamate highlights a vibration calculated to occur at 1078 cm^{-1}. Grey, white, yellow, blue, red, and green spheres represent C, H, S, N, O, and Mg atoms, respectively.

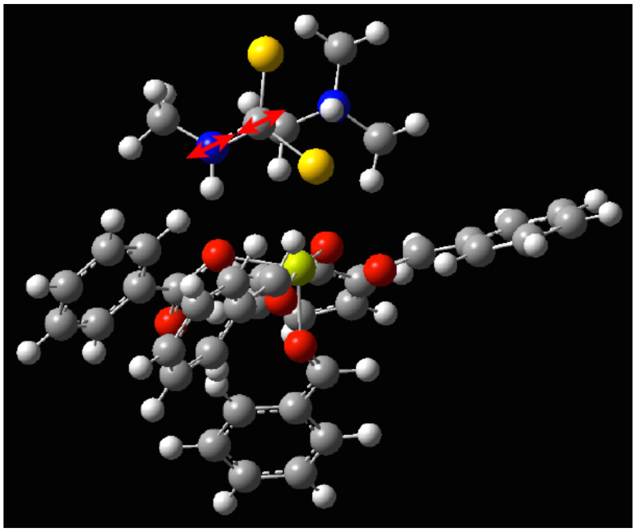

Supplementary Figure 59 | Vibration calculation of geometrically optimized truncated single-site model of CS_2-dosed mm-2–Mg_2(dobpdc). The two red arrows on the carbon and nitrogen of the dithiocarbamate highlight a vibration calculated to occur at 1420 cm^{-1}. Grey, white, yellow, blue, red, and green spheres represent C, H, S, N, O, and Mg atoms, respectively.

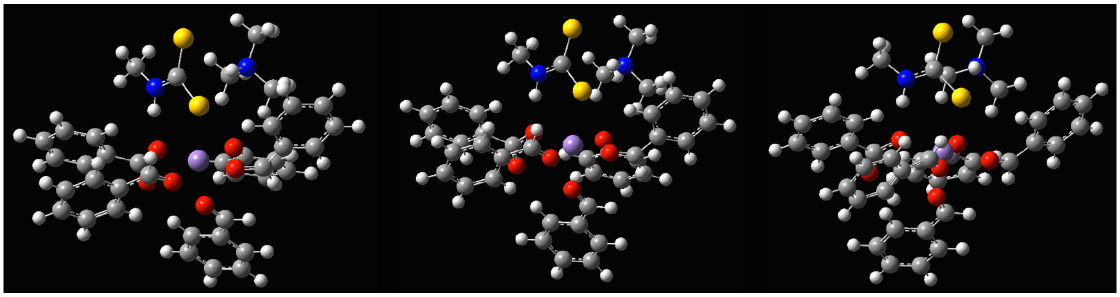

Supplementary Figure 60 | Geometrically optimized truncated single-site model of CS_2-dosed mm-2–Mn_2(dobpdc). All three images are of the same model from different angles. Grey, white, yellow, blue, red, and purple spheres represent C, H, S, N, O, and Mn atoms, respectively.

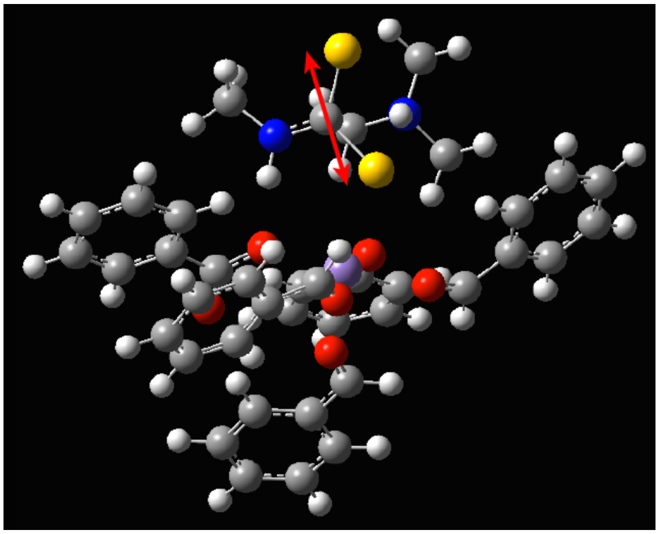

Supplementary Figure 61 | Vibration calculation of geometrically optimized truncated single-site model of CS_2-dosed mm-2–Mn_2(dobpdc). The red arrow on the carbon of the dithiocarbamate highlights a vibration calculated to occur at 943 cm^{-1}. Grey, white, yellow, blue, red, and purple spheres represent C, H, S, N, O, and Mn atoms, respectively.

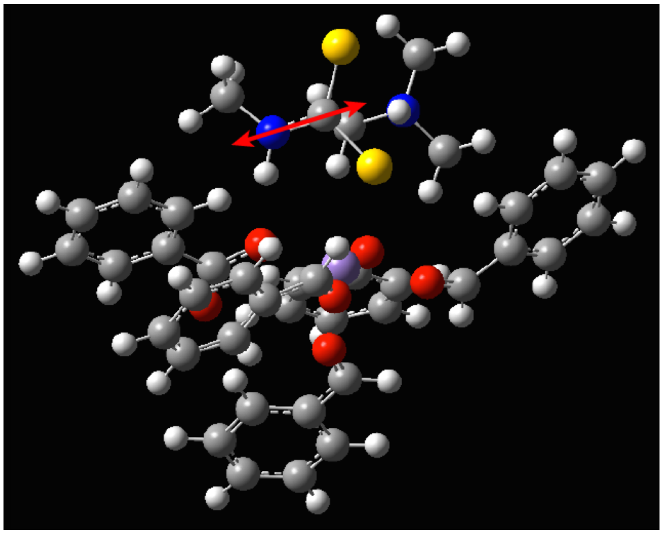

Supplementary Figure 62 | Vibration calculation of geometrically optimized truncated single-site model of CS_2-dosed mm-2–Mn_2(dobpdc). The red arrow on the carbon and nitrogen of the dithiocarbamate highlights a vibration calculated to occur at 1073 cm^{-1}. Grey, white, yellow, blue, red, and purple spheres represent C, H, S, N, O, and Mn atoms, respectively.

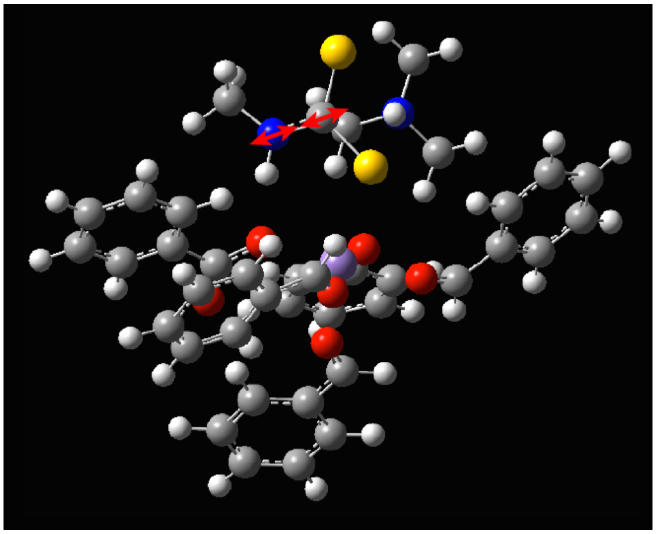

Supplementary Figure 63 | Vibration calculation of geometrically optimized truncated single-site model of CS_2-dosed mm-2–Mn_2(dobpdc). The two red arrows on the carbon and nitrogen of the dithiocarbamate highlight a vibration calculated to occur at 1412 cm^{-1}. Grey, white, yellow, blue, red, and purple spheres represent C, H, S, N, O, and Mn atoms, respectively.

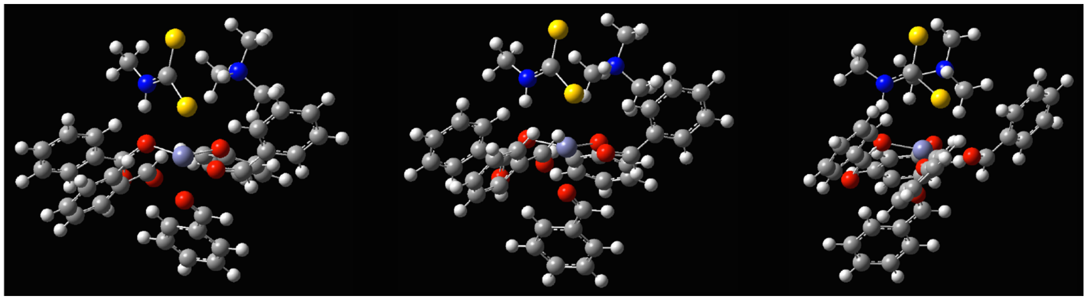

Supplementary Figure 64 | Geometrically optimized truncated single-site model of CS_2-dosed mm-2–Zn_2(dobpdc). All three images are of the same model from different angles. Grey, white, yellow, blue, red, and dark purple spheres represent C, H, S, N, O, and Zn atoms, respectively.

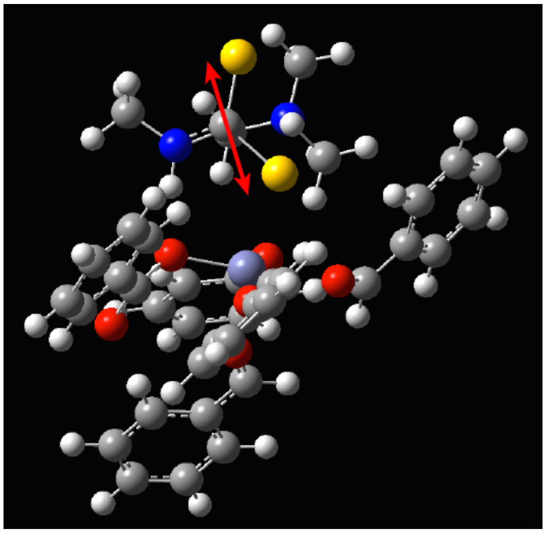

Supplementary Figure 65 | Vibration calculation of geometrically optimized truncated single-site model of CS_2-dosed mm-2–Zn_2(dobpdc). The red arrow on the carbon of the dithiocarbamate highlights a vibration calculated to occur at 945 cm^{-1}. Grey, white, yellow, blue, red, and dark purple spheres represent C, H, S, N, O, and Zn atoms, respectively.

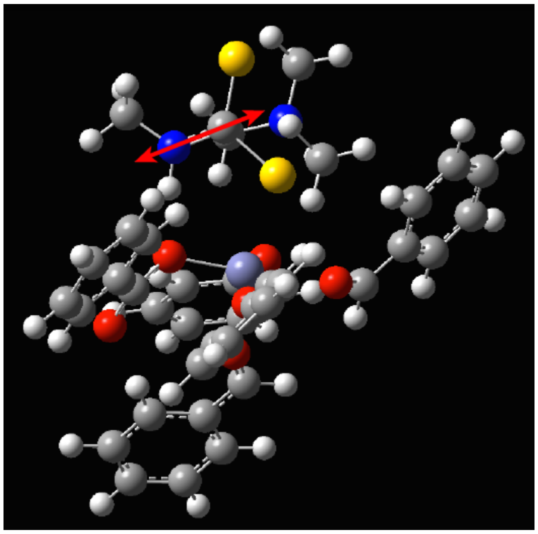

Supplementary Figure 66 | Vibration calculation of geometrically optimized truncated single-site model of CS_2-dosed mm-2–Zn_2(dobpdc). The red arrow on the carbon and nitrogen of the dithiocarbamate highlights a vibration calculated to occur at 1082 cm^{-1}. Grey, white, yellow, blue, red, and dark purple spheres represent C, H, S, N, O, and Zn atoms, respectively.

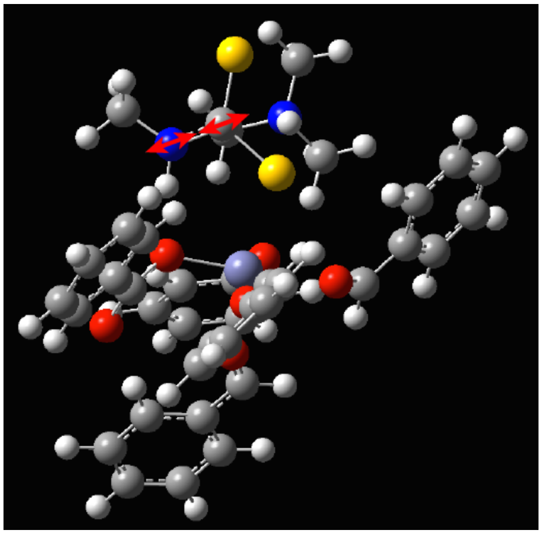

Supplementary Figure 67 | Vibration calculation of geometrically optimized truncated single-site model of CS_2-dosed mm-2–Zn_2(dobpdc). The two red arrows on the carbon and nitrogen of the dithiocarbamate highlight a vibration calculated to occur at 1420 cm^{-1}. Grey, white, yellow, blue, red, and dark purple spheres represent C, H, S, N, O, and Zn atoms, respectively.

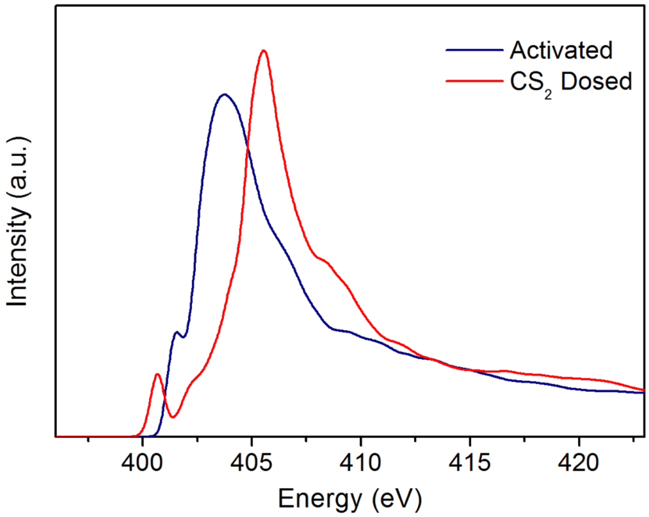

Supplementary Figure 68 | Simulated N K-edge spectra of activated mm-2–Mg_2(dobpdc) (blue) and CS_2-dosed (red) mm-2–Mg_2(dobpdc) at 300 K. An ammonium diothiocarbamate chain structure was used for the calculations, and the main empirically observed features are reproduced.

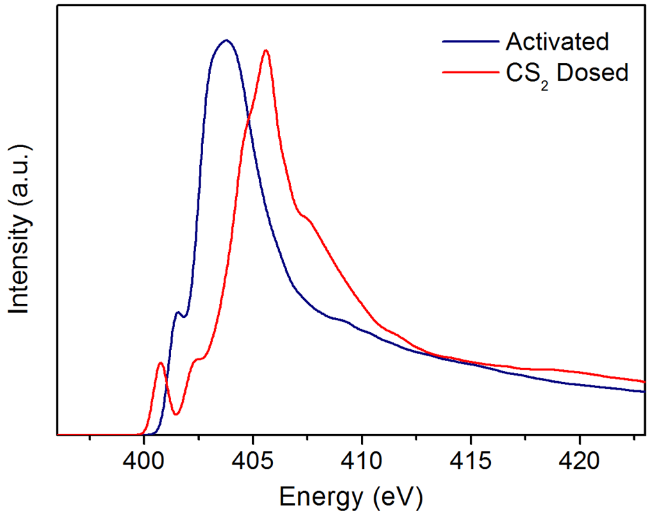

Supplementary Figure 69 | Simulated N K-edge spectra of activated mm-2–Zn_2(dobpdc) (blue) and CS_2-dosed (red) mm-2–Zn_2(dobpdc) at 300 K. An ammonium diothiocarbamate chain structure was used for the calculations, and the main empirically observed features are reproduced.

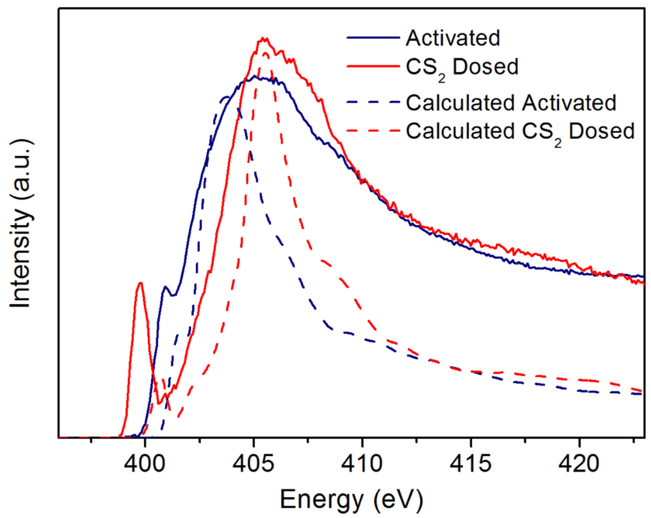

Supplementary Figure 70 | Overlay of experimental and simulated N K-edge spectra of activated mm-2–Mg_2(dobpdc) (blue) and CS_2-dosed (red) mm-2–Mg_2(dobpdc). Solid line = experiment, dash line = calculation at 300 K. An ammonium diothiocarbamate chain structure was used for the calculations, and the main empirically observed features are reproduced.

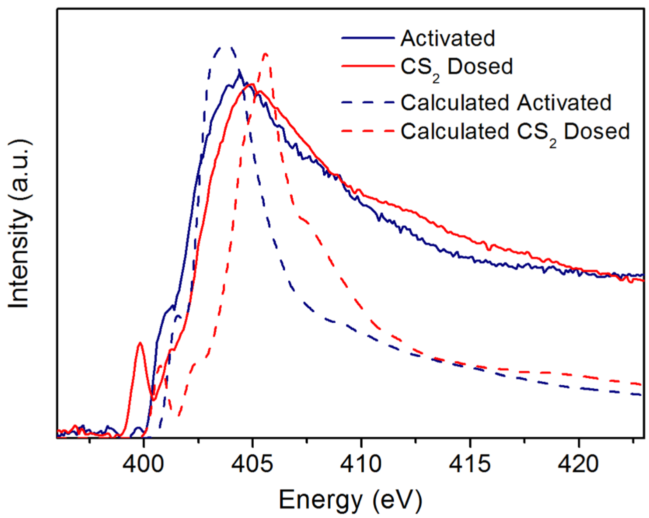

Supplementary Figure 71 | Overlay of experimental and simulated N K-edge spectra of activated mm-2–Zn_2(dobpdc) (blue) and CS_2-dosed (red) mm-2–Zn_2(dobpdc) at 300 K. Solid line = experiment, dash line = calculation at 300 K. An ammonium diothiocarbamate chain structure was used for the calculations, and the main empirically observed features are reproduced.

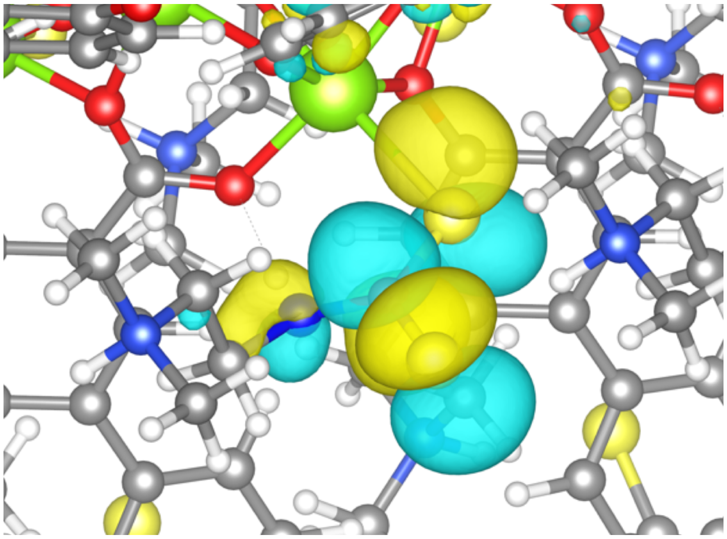

Supplementary Figure 72 | Density distribution of the electronic component of the core-excited final-state orbital at 400.6 eV, corresponding to the pre-edge dithiocarbamate π* transition found in the 0 K DFT calculated N K-edge CS_2-dosed mm-2–Mg_2(dobpdc) spectrum. Grey, white, yellow, blue, red, and green spheres represent C, H, S, N, O, and Mg atoms, respectively.

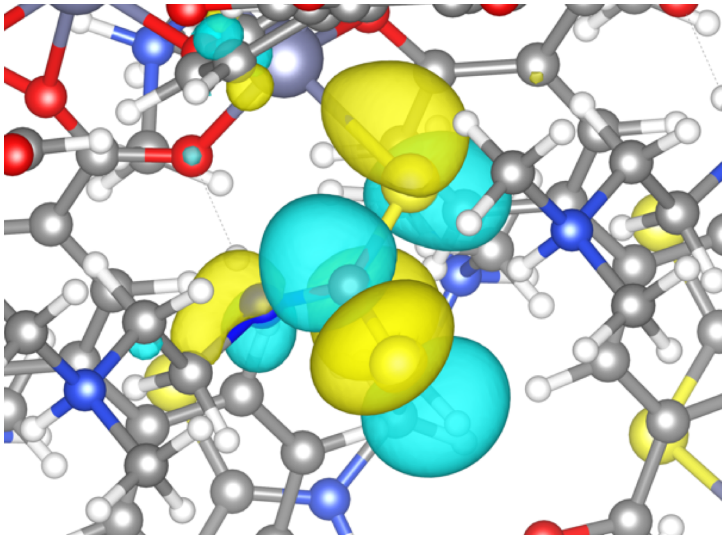

Supplementary Figure 73 | Density distribution of the electronic component of the core-excited final-state orbital at 400.7 eV, corresponding to the pre-edge dithiocarbamate π* transition found in the 0 K DFT calculated N K-edge CS_2-dosed mm-2–Zn_2(dobpdc) spectrum. Grey, white, yellow, blue, red, and purple spheres represent C, H, S, N, O, and Zn atoms, respectively.

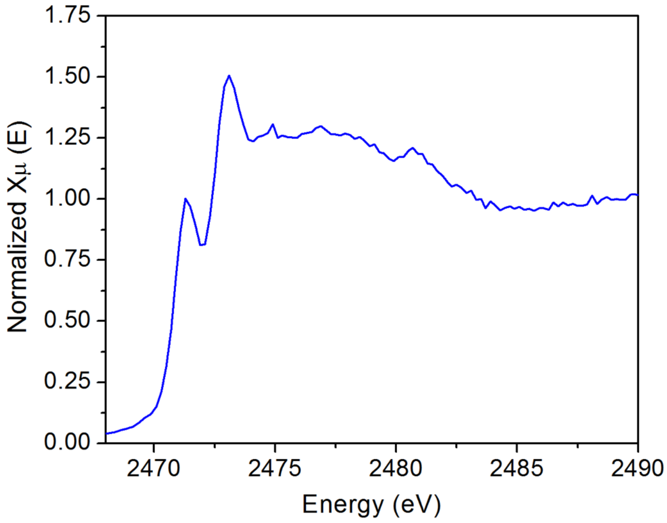

Supplementary Figure 74 | Experimental S K-edge spectrum of *ex situ* CS_2-dosed mm-2–Mg_2(dobpdc).

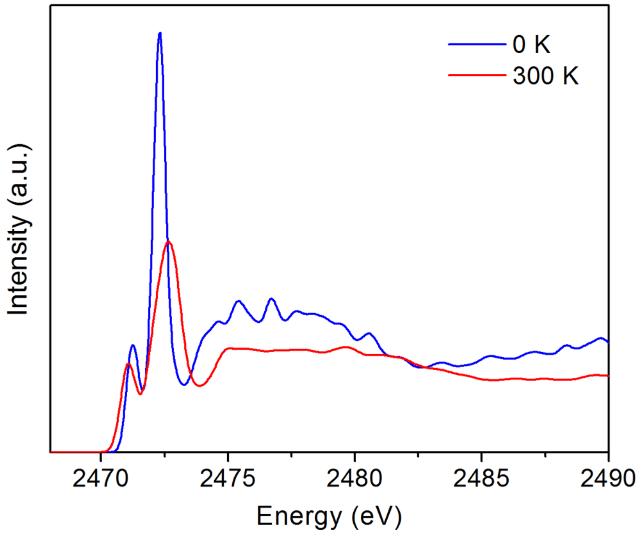

Supplementary Figure 75 | Comparison of computational methods for simulating the S K-edge spectrum of *ex situ* CS_2-dosed mm-2–Mg_2(dobpdc). Comparison of simulated ammonium dithiocarbamate chain spectrum at 0 K (blue line) to *ab initio* molecular dynamics simulations at 300 K (red line). The main features of the empirical spectrum are reproduced in both methods.

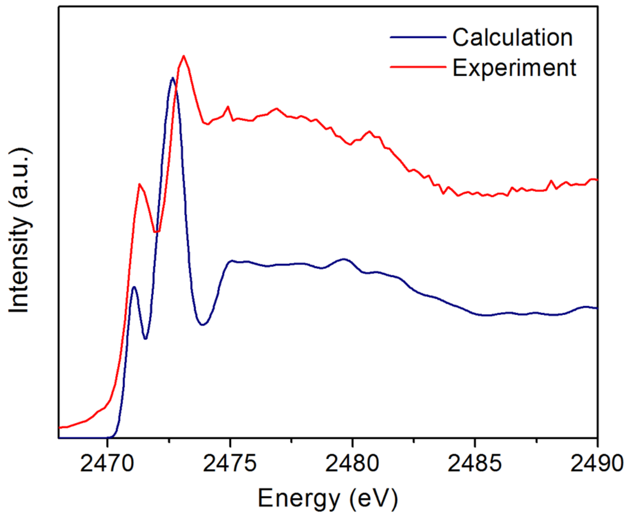

Supplementary Figure 76 | Comparison of calculation and experiment for S K-edge spectrum of *ex situ* CS_2-dosed mm-2–Mg_2(dobpdc). Comparison of simulated ammonium dithiocarbamate chain spectrum at 300 K (blue line) to experimental data (red line). The main features of the empirical spectrum are reproduced in the simulated spectrum.

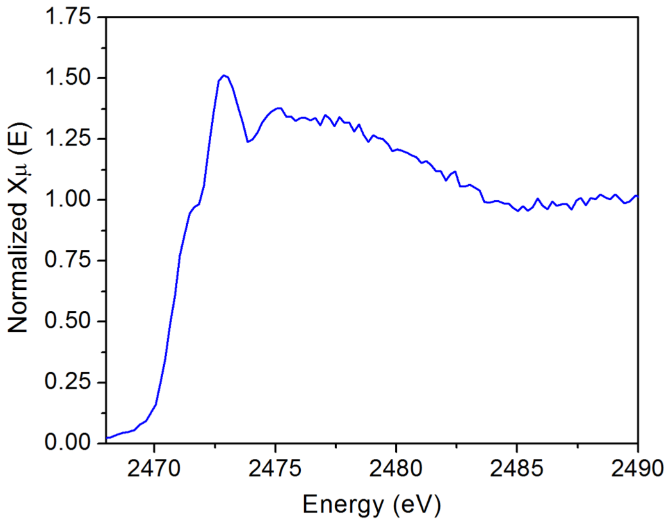

Supplementary Figure 77 | Experimental S K-edge spectrum of *ex situ* CS_2-dosed mm-2–Zn_2(dobpdc).

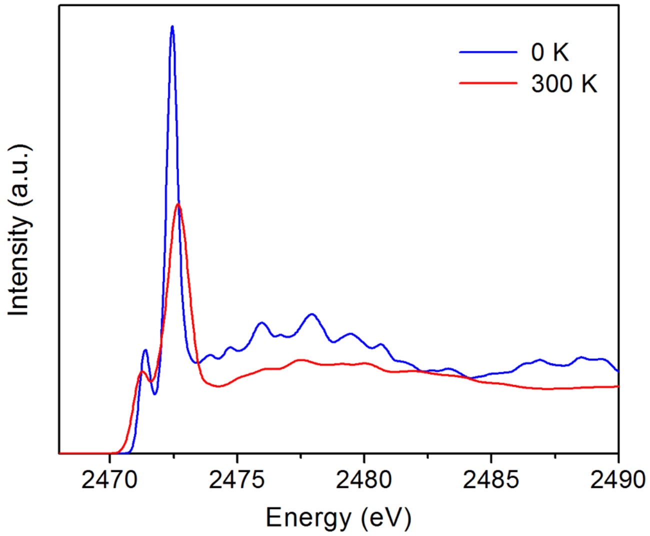

Supplementary Figure 78 | Comparison of computational methods for simulating the S K-edge spectrum of *ex situ* CS_2-dosed mm-2–Zn_2(dobpdc). Comparison of simulated ammonium dithiocarbamate chain spectrum at 0 K (blue line) to *ab initio* molecular dynamics simulations at 300 K (red line). The main features of the empirical spectrum are reproduced in both methods.

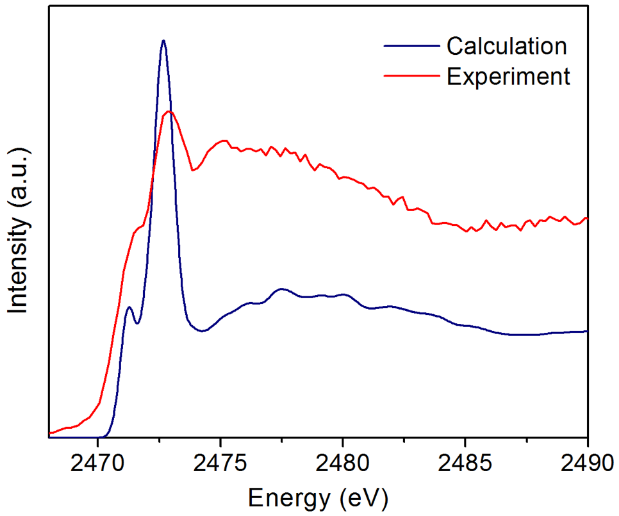

Supplementary Figure 79 | Comparison of calculation and experiment for S K-edge spectrum of *ex situ* CS_2-dosed mm-2–Zn_2(dobpdc). Comparison of simulated ammonium dithiocarbamate chain spectrum at 300 K (blue line) to experimental data (red line). The main features of the empirical spectrum are reproduced in the simulated spectrum.

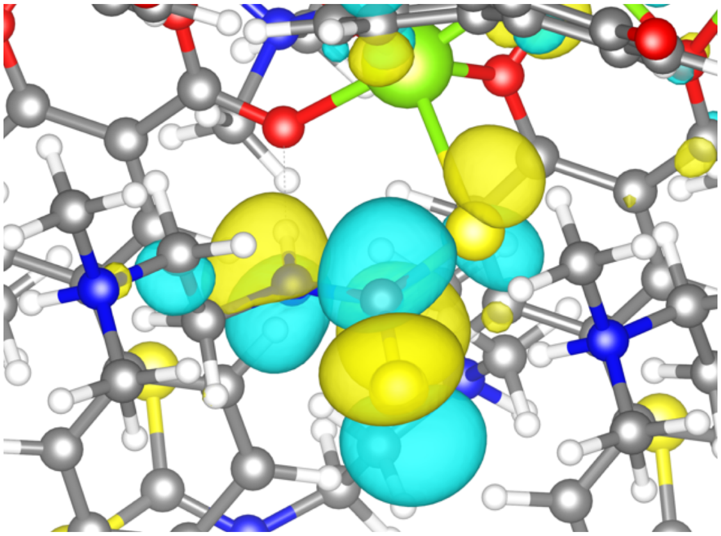

Supplementary Figure 80 | Density distribution of the electronic component of the core-excited final-state orbital at 2471.1 eV, corresponding to the pre-edge dithiocarbamate π* transition found in the 0 K DFT calculated S K-edge CS_2-dosed mm-2–Mg_2(dobpdc) spectrum. Grey, white, yellow, blue, red, and green spheres represent C, H, S, N, O, and Mg atoms, respectively.

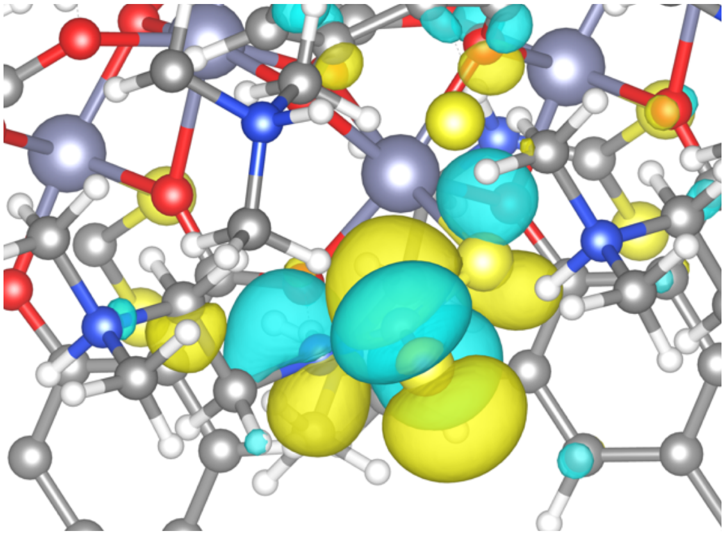

Supplementary Figure 81 | Density distribution of the electronic component of the core-excited final-state orbital at 2471.3 eV, corresponding to the pre-edge dithiocarbamate π* transition found in the 0 K DFT calculated S K-edge CS_2-dosed mm-2–Zn_2(dobpdc) spectrum. Grey, white, yellow, blue, red, and purple spheres represent C, H, S, N, O, and Zn atoms, respectively.

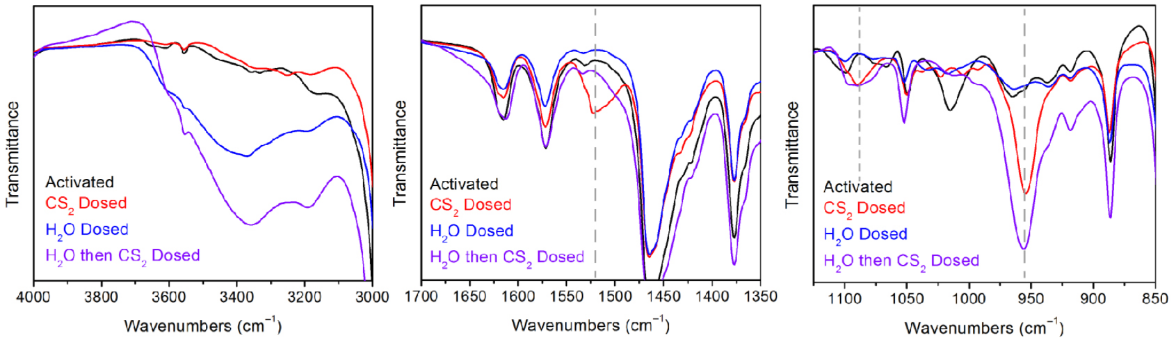

Supplementary Figure 82 | Transmittance mode FT–IR Spectra of mm-2–Mg_2(dobpdc). Black line = activated, red line = CS_2 dosed for 10 minutes, blue line = H_2O dosed for 16 hours, and purple line = H_2O dosed for 16 hours, then CS_2 dosed for 10 minutes. Dashed lines highlight bands arising from C−S vibrations in CS_2 dosed samples.

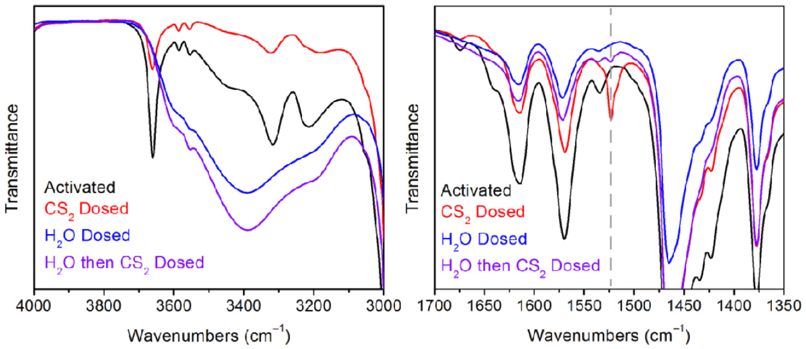

Supplementary Figure 83 | Transmittance mode FT–IR Spectra of Mg_2(dobpdc). Black line = activated, red line = CS_2 dosed for 10 minutes, blue line = H_2O dosed for 16 hours, and purple line = H_2O dosed for 16 hours, then CS_2 dosed for 60 minutes. Dashed lines highlight bands arising from C−S vibrations in CS_2 dosed samples.

Supplementary References

1) Waddington, G., Smith, J. C., Williamson, K. D. & Scott, D. W. Carbon disulfide as a reference substance for vapor-flow calorimetry; the chemical thermodynamic properties. *J. Phys. Chem.* **66**, 1074-1077 (1962).
2) Siegelman, R. L. *et al.* Controlling cooperative CO_2 adsorption in diamine-appended Mg_2(dobpdc) metal-organic frameworks. *J. Am. Chem. Soc.* **139**, 10526-10538 (2017).
3) *SAINT*, *APEX2*, and *APEX3* software for CCD diffractometers (Bruker Analytical X-ray Systems Inc., 2014).
4) Sheldrick, G. M. *SADABS* (University of Gottingen: Gottingen, Germany, 1996).
5) Sheldrick, G. M. Crystal structure refinement with *SHELXL*. *Acta Crystallogr. C Struct. Chem.* **71**, 3-8 (2015).
6) Dolomanov, O. V., Bourhis, L. J., Gildea, R. J., Howard, J. A. K. & Puschmann, H. *OLEX2*: A complete structure solution, refinement and analysis program. *J. Appl. Crystallogr.* **42**, 339-341 (2009).
7) Bruker AXS, TOPAS, version 4.1. (2007).
8) Pawley, G. S. Unit-cell refinement from powder diffraction scans. *J. Appl. Cryst.* **14**, 357-361 (1981).
9) Rietveld, H. M. A profile refinement method for nuclear and magnetic structures. *J. Appl. Cryst.* **2**, 65-71 (1969).
10) Pimentel, G. C. & McClellan, A. L. The hydrogen bond. (1960).
11) Plyler, E. K. & Humphreys, C. Infrared absorption spectrum of carbon disulfide. *J. Res. Natl. Bur. Stand.* **39**, 59-65 (1947).
12) Peverati, R. & Truhlar, D. G. M11-L: A local density functional that provides improved accuracy for electronic structure calculations in chemistry and physics. *J. Phys. Chem. Lett.* **3**, 117-124 (2011).
13) Gaussian. *Pseudo* <http://gaussian.com/pseudo/> (2017).
14) Drisdell, W. S. & Kortright, J. B. Gas cell for in situ soft X-ray transmission-absorption spectroscopy of materials. *Rev. Sci. Instrum.* **85**, 074103 (2014).
15) Drisdell, W. S. *et al.* Probing adsorption interactions in metal-organic frameworks using X-ray spectroscopy. *J. Am. Chem. Soc.* **135**, 18183-18190 (2013).
16) Drisdell, W. S. *et al.* Probing the mechanism of CO_2 capture in diamine-appended metal-organic frameworks using measured and simulated X-ray spectroscopy. *Phys. Chem. Chem. Phys.* **17**, 21448-21457 (2015).
17) Ravel, B. & Newville, M. *ATHENA*, *ARTEMIS*, *HEPHAESTUS*: Data analysis for X-ray absorption spectroscopy using *IFEFFIT*. *J. Synchrotron. Radiat.* **12**, 537-541 (2005).
18) Marcus, M. A. *et al.* Beamline 10.3.2 at ALS: A hard X-ray microprobe for environmental and materials sciences. *J. Synchrotron. Radiat.* **11**, 239-247 (2004).
19) Kresse, G. & Furthmuller, J. Efficiency of ab-initio total energy calculations for metals and semiconductors using a plane-wave basis set. *Comput. Mater. Sci.* **6**, 15-50 (1996).
20) Kresse, G. & Furthmuller, J. Efficient iterative schemes for ab initio total-energy calculations using a plane-wave basis set. *Phys. Rev. B* **54**, 11169-11186 (1996).
21) Kohn, W. & Sham, L. J. Self-consistent equations including exchange and correlation effects. *Phys. Rev.* **140**, A1133-A1138 (1965).
22) Perdew, J. P., Burke, K. & Ernzerhof, M. Generalized gradient approximation made simple. *Phys. Rev. Lett.* **77**, 3865–3868 (1996).

23) Blochl, P. E. Projector augmented-wave method. *Phys. Rev. B.* **50**, 17953-17979 (1994).
24) Kresse, G. & Joubert, D. From ultrasoft pseudopotentials to the projector augmented-wave method. *Phys. Rev. B.* **59**, 1758-1775 (1999).
25) Lee, K., Murray, É. D., Kong, L., Lundqvist, B. I. & Langreth, D. C. Higher-accuracy van der Waals density functional. *Phys. Rev. B* **82** (2010).
26) Harl, J., Schimka, L. & Kresse, G. Assessing the quality of the random phase approximation for lattice constants and atomization energies of solids. *Phys. Rev. B* **81** (2010).
27) Kresse, G. & Hafner, J. Ab initio molecular dynamics for liquid metals. *Phys. Rev. B.* **47**, 558-561 (1993).
28) Nosé, S. A unified formulation of the constant temperature molecular dynamics methods. *J. Chem. Phys.* **81**, 511-519 (1984).
29) Giannozzi, P. *et al.* Quantum Espresso: A modular and open-source software project for quantum simulations of materials. *J. Phys. Condens. Matter* **21**, 395502 (2009).
30) Vanderbilt, D. Soft self-consistent pseudopotentials in a generalized eigenvalue formalism. *Phys. Rev. B* **41**, 7892-7895 (1990).
31) Prendergast, D. & Galli, G. X-ray absorption spectra of water from first principles calculations. *Phys. Rev. Lett.* **96**, 215502 (2006).
32) Stohr, J. *NEXAFS spectroscopy*. (Springer, 1992).
33) Cohen, A. J., Mori-Sánchez, P. & Yang, W. Fractional charge perspective on the band gap in density-functional theory. *Phys. Rev. B* **77**, 115123 (2008).
34) Mori-Sanchez, P., Cohen, A. J. & Yang, W. Localization and delocalization errors in density functional theory and implications for band-gap prediction. *Phys. Rev. Lett.* **100**, 146401 (2008).
